# Mapping the widespread distribution and transmission dynamics of linezolid resistance in humans, animals, and the environment

**DOI:** 10.1186/s40168-023-01744-2

**Published:** 2024-03-13

**Authors:** Weiyi Shen, Chang Cai, Ning Dong, Jiawei Chen, Rong Zhang, Jiachang Cai

**Affiliations:** 1 Clinical Microbiology Laboratory, The Second Affiliated Hospital of Zhejiang University School of Medicine, Zhejiang University, Hangzhou, China; 2 China Australia Joint Laboratory for Animal Health Big Data Analytics, College of Animal Science and Technology, Zhejiang Agricultural and Forestry University, Hangzhou, China; 3 Department of Medical Microbiology, School of Biology and Basic Medical Science, Medical College of Soochow University, Suzhou, China; 4 Suzhou Key Laboratory of Pathogen Bioscience and Anti-Infective Medicine, Soochow University, Suzhou, China

**Keywords:** Linezolid resistance, Human, Animal, Environment, *optrA*, One health

## Abstract

**Background:**

The rise of linezolid resistance has been widely observed both in clinical and non-clinical settings. However, there were still data gaps regarding the comprehensive prevalence and interconnections of linezolid resistance genes across various niches.

**Results:**

We screened for potential linezolid resistance gene reservoirs in the intestines of both humans and animals, in meat samples, as well as in water sources. A total of 796 bacteria strains out of 1538 non-duplicated samples were identified to be positive for at least one linezolid resistance gene, *optrA*, *poxtA*, *cfr*, and *cfr*(D). The prevalence of *optrA* reached 100% (95% CI 96.3–100%) in the intestines of pigs, followed by fish, ducks, and chicken at 77.5% (95% CI 67.2–85.3%), 62.0% (95% CI 52.2–70.9%), and 61.0% (95% CI 51.2–70.0%), respectively. The meat and water samples presented prevalences of 80.0% (95% CI 70.6–87.0%) and 38.0% (95% CI 25.9–51.9%), respectively. The unreported prevalence of the *cfr*(D) gene was also relatively higher at 13.0% (95% CI 7.8–21.0%) and 19.0% (95% CI 10.9–25.6%) for the feces samples of ducks and pigs, respectively. Enterococci were the predominant hosts for all genes, while several non-enterococcal species were also identified. Phylogenetic analysis revealed a significant genetic distance among linezolid resistance gene reservoirs, with polyclonal structures observed in strains within the same niche. Similar genetic arrays harboring assorted insertion sequences or transposons were shared by reservoirs displaying heterogeneous backgrounds, though large diversity in the genetic environment of linezolid resistance genes was also observed.

**Conclusions:**

The linezolid resistance genes were widespread among various niches. The horizontal transfer played a crucial role in driving the circulation of linezolid resistance reservoirs at the human-animal-environment interfaces.

Video Abstract

**Supplementary Information:**

The online version contains supplementary material available at 10.1186/s40168-023-01744-2.

## Background

The increasing occurrence of antimicrobial resistance (AMR) is currently recognized as a global public health crisis. This issue expands beyond healthcare facilities, as interconnected human, animal, and environmental habitats collectively contribute to the emergence, evolution, and spread of AMR [1]. Consequently, a coordinated and multisectoral approach, such as One Health, has become imperative to comprehend the role of interconnected ecosystems in the complex dynamics of AMR and effectively address the resistance problem [2, 3]. From this perspective, tracking the circulation of AMR genes at the human-animal-environment interface holds profound significance and is considered one of the pivotal issues in One Health [4], particularly concerning the genes that bestow resistance to clinically important antibiotics, such as colistin, carbapenem, vancomycin, and oxazolidinones [5].

Linezolid, the pioneering member of the oxazolidinone family, stands as one of the few agents that remain effective against multidrug-resistant Gram-positive bacteria, including methicillin-resistant *Staphylococcus aureus* (MRSA) and vancomycin-resistant enterococci (VRE) [6]. However, in recent years, the emergence of linezolid resistance in Gram-positive communities worldwide has significantly compromised the clinical utility of this antimicrobial agent. Resistance to linezolid is commonly mediated by mutations in domain V of 23 s rRNA [7], modifications in ribosomal proteins L3 and L4 [8], or acquisition of transferable resistance determinants [9]. These determinants include *optrA*, *poxtA*, *cfr*, as well as its variants. The *cfr* gene [10] and *cfr*-like genes, specifically designated as *cfr*(B) [11], *cfr*(C) [12], *cfr*(D) [13], and *cfr*(E) [14], confer cross-resistance to oxazolidinones, phenicols, lincosamides, pleuromutilins, and streptogramin A (PhLOPS_A_ phenotype) by inhibiting bacterial protein biosynthesis [15]. To date, members of the *cfr* family have been detected in at least two genera of Gram-positive bacteria, except for *cfr*(C) and *cfr*(E), which have been exclusively described in *Clostridium* and/or *Campylobacter* [9].

The novel plasmid-borne *optrA* and *poxtA* genes encode ATP-binding cassette (ABC)-F proteins that confer either resistance or decreased susceptibility to oxazolidinones and phenicols through a ribosomal protection mechanism [16, 17]. Since the identification of the *optrA* gene in enterococci of both human and animal origin in 2015 [16], it has subsequently been detected in various bacteria, including *Staphylococcus*, *Streptococcus*, *Clostridium*, *Campylobacter*, *Aerococcus*, *Lactococcus*, and *Vagococcus* [9, 18–21], exhibiting a remarkable diversity of nucleotide sequences [9, 22, 23]. Initially described in a clinical MRSA in 2018 [17], the *poxtA* gene has also been identified in *Enterococcus* and *Ligilactobacillus* [24]. Recently, a variant of the *poxtA* gene, *poxtA2*, has been discovered to be co-location with the *cfr*(D) gene in a conjugative plasmid of enterococci [25].

Previous studies have demonstrated that linezolid resistance genes are embedded into a plethora of variable genetic environments, involving multiple mobile genetic elements (MGEs). These elements play a crucial role in facilitating the horizontal transfer and further dissemination of these genes across strains, species, genus boundaries, or various potential niches [21, 23, 26–29]. Although linezolid has thus far been exclusively approved for human use, the *cfr*, *optrA*, and *poxtA* genes have been reported to drive the proliferation of linezolid resistance in Gram-positive bacteria within healthcare settings (22, 30–34), among animals and their products [35–45], as well as in various environmental contexts [9, 46–51]. This underscores the significance of monitoring these linezolid resistance determinants through the One Health approach.

Despite concerted efforts to gain a better understanding of the prevalence of linezolid resistance in numerous countries, existing studies generally concentrate on single or sporadic One Health sectors or the occurrence in specific genera, primarily enterococci. Considering the promiscuous nature of MGEs and conjugative plasmids, the rapid dissemination of the resistance locus is highly plausible, and the prevalence is still likely to be underestimated. In 2022, we reported an optimized screening approach with higher sensitivity, resulting in a higher prevalence of the *optrA* gene in fecal samples among healthy individuals at 19.3% in Hangzhou, China [52]. However, there are still data gaps concerning the epidemiological situation of these genes in Gram-positive communities from various niches and the relationships between these linezolid resistance reservoirs. In this study, we aimed to gain comprehensive knowledge of epidemiologic and genetic levels for linezolid resisnce among different niches and depict the potential routes of the transmissions through the One Health approach.

## Methods

### Sample collection and bacterial isolation

A total of 1538 samples were collected from Hangzhou City in China, spanning the period from February to December 2022. The samples encompassed various sources, including feces from healthy individuals (*n* = 1018), feces from farmed animals (100 each for chicken, duck, and pig), fish samples (*n* = 80), pork and poultry meat obtained from the supermarkets (*n* = 90), as well as water samples from five rivers and lakes in Hangzhou City (*n* = 50) (refer to Supplementary Table S1 for details).

As part of our research, we isolated 245 strains from 1018 human feces in our previous study [52]. The fecal samples for pigs, ducks, and chickens were collected using cloacal swabs. The fecal samples for fish were obtained in the form of intestinal content samples. Non-duplicated samples were collected. For meat samples, a standardized protocol was followed, wherein 50 g of meat from each sample was thoroughly vortexed with 40 mL of 0.1 M phosphate buffer. Subsequently, the mixture was homogenized to ensure accurate representation. In the case of water samples, a meticulous procedure was employed, wherein 500 mL of water was filtered through 0.45 µm filter membranes (Pall, USA). These filter membranes were then subjected to three rounds of washing with 10 mL of sterile saline, ensuring optimal sample preparation. The acquired liquid products were then combined and mixed. The isolation of Gram-positive cocci carrying linezolid resistance genes was conducted using our optimized approach, as previously described [52]: pretreated products and feces samples were inoculated into 5 ml of Luria–Bertani (LB) broth and incubated at 37 °C for 24 h. Subsequently, 100 μl of each enrichment was transferred to a subculture of 5 ml fresh LB broth containing 5% NaCl and 10 mg/L florfenicol. The Columbia agar base media, supplemented with 5% (v/v) sheep blood and 10 mg/L of florfenicol, was used to select and purify the putative target isolates.

### Species identification and PCR analysis

Species identification was performed using MALDI-TOF MS (Bruker Daltonik GmbH, Germany) with the Biotyper Version 3.0 (DB2969 database). Additionally, confirmation of species identification was obtained by comparing the isolates with reference strains through the online tool Average Nucleotide Identity (ANI) calculator (http://enve-omics.ce.gatech.edu/ani/index) for those isolates that underwent WGS. Subsequently, isolates determined to be of the same species according to DB2969 database (including *Enterococcus dongliensis*, *E. hulanensis*, *E. lactis*, *E. viikkiensis*, and *E. xiangfangensis*) were employed to construct an in-house database. This in-house database was then utilized for the re-confirmation of non-sequenced enterococci, ensuring accuracy and reliability in the species identification process.

The presence of *optrA*, *poxtA*, *cfr*, and *cfr*-like genes was ascertained through PCR analysis and Sanger sequencing. To identify OptrA variants, a comparison was made between the deduced amino acid sequences of the isolates and the original OptrA from *E. faecalis* E349, which had been previously designated as the wild type [16].

### Antimicrobial susceptibility testing

The minimal inhibitory concentrations (MICs) of seven antimicrobial agents, namely linezolid, chloramphenicol, penicillin G, ciprofloxacin, erythromycin, tetracycline, and vancomycin, were determined using the broth microdilution method [53]. The interpretation of results was conducted in accordance with the standard of Clinical and Laboratory Standards Institute (CLSI) [54, 55]. For the *Vagococcus* and *Globicatella* genera, the susceptibility breakpoints of the seven antimicrobial agents, as established for enterococci, were applied. To ensure the accuracy and reliability of the analysis, *E. faecalis* ATCC 29212, *S. aureus* ATCC 29213, and *Streptococcus pneumoniae* ATCC 49619 were employed as the quality control strains.

### Whole-genome sequencing and genome analysis

The cohort selection of isolates subjected to WGS was carried out through a systematic approach, involving stratification based on their sources, species, and carriage of linezolid resistance genes. All isolates identified as non-enterococcal species underwent sequencing. Additionally, all enterococci carrying any of the *poxtA*, *cfr*, or *cfr*-like genes were also subjected to WGS. To manage the considerable number of enterococci exclusively carrying *optrA*, 10% of strains from each species within each niche were randomly selected using a Microsoft Excel randomization program for WGS. This approach resulted in a final cohort of 294 isolates for WGS, comprising 208 enterococci and 86 non-enterococcal ones (Supplementary Table S1).

The genomic DNA underwent WGS utilizing the Illumina NovaSeq 6000 platform. Subsequently, the sequencing data were de novo assembled using SPAdes Version 3.13.1 [56]. The identification of antimicrobial resistance genes (ARGs) and the sequence types (STs) for the assembly scaffolds were performed with default settings, utilizing ResFinder Version 4.1 [57] and MLST Version 2.0 [58], respectively. The newly obtained STs were uploaded and assigned through PubMLST (https://pubmlst.org/). Furthermore, the 16S rRNA genes of all isolates were obtained using Barrnap Version 0.9 (https://github.com/tseemann/barrnap). The alignment of the 16S sequences was conducted using MAFFT Version 7 [59]. For the construction of the maximum likelihood phylogenetic tree based on the 16S rRNA of all strains subjected to WGS, IQ-TREE Version 1.6.12 was employed [60]. To facilitate core-genome alignment, single-nucleotide polymorphism (SNP) calling, and the construction of SNP-based phylogenetic trees, the Parsnp script [61] was utilized. Subsequently, all the phylogenetic trees were visualized presented and annotated using the online tool iTOL Version 3 [62]. BLASTN analysis was performed to compare and annotate the contigs containing the target genes with known sequences of the NCBI database. The sequences of strains with short linezolid resistance gene-carrying contigs were mapped to the known structures in this study, and the potential gaps between contigs were filled by PCR analysis and Sanger sequencing to generate complete structures. Additionally, the linear alignment of the genetic environment of the linezolid resistance genes in different isolates was visualized using EasyFig Version 2.2.2 [63].

## Results

### Prevalence and distribution of linezolid resistance genes in various niches

The prevalence and distribution of linezolid resistance genes were illustrated in Table 1. A total of 796 florfenicol-resistant strains were obtained, comprising 245 isolates of human origin, 78 isolates from meat, 74 isolates from cloacal swabs of chicken, 102 isolates from duck feces, 191 isolates from pig feces, and 24 isolates from water. The *optrA* gene was most frequently detected among each niche, with an overall prevalence of 37.2% (95% CI 34.8–39.6%). The highest prevalence of *optrA* was exhibited in the intestine of pigs (100.0%, 95% CI 96.3–100%), followed by meat samples (80.0%, 95% CI 70.6–87.0%). Among fish samples, a prevalence of 77.5% (95% CI 67.2–85.3%) was observed, which was about twice that for water samples at 38.0% (95% CI 25.9–51.9%). The fish showed the highest prevalence of the *poxtA* gene at 15.0% (95% CI 8.8–24.4%), followed by pigs at 12.0% (95% CI 7.0–19.8%). In water, the prevalence was 8.0% (95% CI 3.2–18.8%). The *cfr* gene was mainly detected in isolates from pig feces at 19.0% (95% CI 12.5–27.8%). Likewise, a higher prevalence of the *cfr*(D) gene in the intestine of pigs was observed at 17.0% (95% CI 10.9–25.6%), and an intestinal carriage rate of ducks was also observed at 13.0% (95% CI 7.8–21.0%). The *cfr*(B), *cfr*(C), or *cfr*(E) genes were not identified among these isolates.

**Table 1:** Prevalence and distribution of linezolid resistance genes in various niches

**Source**	**No. of samples**	**Prevalence % (95% CI)**					**Distribution of the *optrA *carriers**							
		***optrA***	***poxtA***	***cfr***	***cfr*(D)**	***cfr*(B/C/E)**	***Enterococcus***	***Lactococcus***	***Vagococcus***	***Streptococcus***	***Aerococcus***	***Globicatella***	**No. of strains**	**No. of samples**
**human faeces**	1018	19.3%(16.95%-21.79%)	2.3%(1.51%-3.37%)	0.3%(3.15%-18.84%)	0.5%(0.21%-1.14%)	0.0%(0%-0.38%)	227	2	1	1	-	-	231	196
**retail meat**	90	80.0%(70.59%-86.96%)	2.2%(0.61%-7.74%)	1.1%(0.2%-6.03%)	4.4%(1.74%-10.87%)	0.0%(0%-4.09%)	76	2	-	-	-	-	78	72
**clonal swab of chicken**	100	61.0%(51.2%-69.98%)	2.0%(0.55%-7.00%)	1.0%(0.18%-5.45%)	4.0%(1.57%-9.84%)	0.0%(0%-3.70%)	72	-	1	-	-	-	73	61
**duck faeces**	100	62.0%(52.21%-70.9%)	6.0%(2.78%-12.48%)	2.0%(0.55%-7.00%)	13.0%(7.76%-20.98%)	0.0%(0%-3.70%)	88	-	6	1	1	-	96	62
**pig faeces**	100	100.0%(96.3%-100%)	12.0%(7.00%-19.81%)	19%(12.51%-27.78%)	17%(10.89%-25.55%)	0.0%(0%-3.70%)	172	5	11	1	-	1	190	100
**intestinal contents of fish**	80	77.5%(67.21%-85.27%)	15.0%(8.79%-24.41%)	0.0%(0%-3.7%)	1.3%(0.22%-6.75%)	0.0%(0%-4.58%)	31	48	2	-	-	-	81	62
**surface water**	50	38.0%(25.86%-51.85%)	8.0%(3.15%-18.84%)	2.0%(0.35%-10.5%)	0.0%(0%-7.13%)	0.0%(0%-7.13%)	23	1	-	-	-	-	24	19
**total**	1538	37.2%(34.81%-39.63%)	4.0%(3.15%-18.84%)	1.8%(1.21%-2.55%)	2.9%(2.14%-3.82%)	0.0%(0%-0.25%)	689	58	21	3	1	1	773	572

**Source**	**Distribution of the *poxtA* carriers**					**Distribution of the *cfr *carriers**			**Distribution of the *cfr*(D) carriers**					**Total No.of florfenicol-resistant strains**
	***Enterococcus***	***Lactococcus***	***Ligilactobacillus***	**No. of strains**	**No. of samples**	***Enterococcus***	**No. of strains**	**No. of samples**	***Enterococcus***	***Vagococcus***	***Streptococcus***	**No. of strains**	**No. of samples**	
**human faeces**	22	-	1	23	23	3	3	3	5	-	-	5	5	245
**retail meat**	2	-	-	2	2	1	1	1	4	-	-	4	4	78
**clonal swab of chicken**	2	-	-	2	2	1	1	1	3	1	-	4	4	74
**duck faeces**	6	-	-	6	6	2	2	2	12	-	1	14	13	102
**pig faeces**	13	1	-	14	12	21	21	19	11	6	-	17	17	191
**intestinal contents of fish**	10	2	-	12	12	-	-	-	1	-	-	1	1	82
**surface water**	4	-	-	4	4	1	1	1	-	-	-	-	-	24
**total**	59	3	1	63	61	29	29	27	36	7	1	45	44	796

Enterococci were the primary hosts for the *optrA* gene in most of the niches, except for fish. A total of 689 *optrA*-positive enterococci of 19 species were collected from various niches (Supplementary Table S1), with *E. faecalis* (*n* = 402), *E. faecium* (*n* = 91), and *E. avium* (*n* = 64) being the most frequently identified species. In contrast, within the intestine of the fish, *Lactococcus lactis* (35/81) was found to be the more prevalent carrier of the *optrA* gene, followed by *E. avium* (17/81) and *L. petauri* (11/81). Additionaly, a variety of *optrA*-carrying non-enterococcal isolates were obtained from diverse niches, including *Lactococcus* (*L. raffinolactis*, *L. garvieae*, and *L. formosensis*), *Vagococcus (Vagococcus lutrae*, *V. fluvialis*, *and V. carniphilus)*, *Streptococcus* (*Streptococcus gallolyticus* and *S. parauberis*), *Aerococcus urinaeequi*, and *Globicatella sulfidifaciens*. Notably, this study marks the first detection of the *optrA* gene in *L. lactis*, *L. raffinolactis*, *V. fluvialis*, *V. carniphilus*, *S. parauberis*, *A. urinaeequi*, and *G. sulfidifaciens*.

Regarding the *poxtA* gene, it was predominantly detected in enterococci, while one *L. lactis* from pig feces and one *L. raffinolactis* of fish origin also tested positive for this gene. The *cfr* gene was only present among enterococci. However, the *cfr*(D) gene was also detected in non-enterococcal isolates including *V. carniphilus* of porcine origin, *V. fluvialis* from chicken, and *S. parauberis* from ducks. All *cfr*- or *cfr*(D)-positive isolates showed concomitant carriage of the *optrA* and/or *poxtA* gene(s), with the exception of a single strain where these genes were absent.

### Antimicrobial susceptibility results

**Fig. 1:**
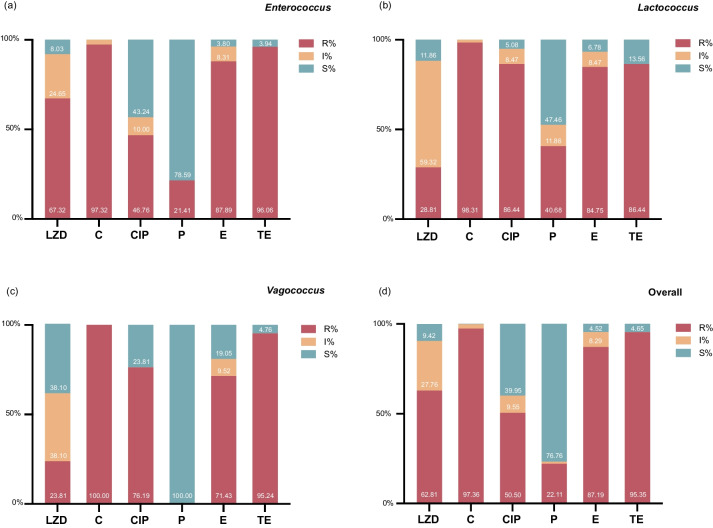
Antimicrobial susceptibility profiles for linezolid resistance gene reservoirs. LZD, linezolid; C, chloramphenicol; CIP, ciprofloxacin; P, penicillin G; E, erythromycin; TE, tetracycline; R, resistance rate; I, intermediate rate; S, susceptibility rate

A similar antimicrobial susceptibility pattern with high percentages of resistance to chloramphenicol, erythromycin, and tetracycline was observed across different genera of isolates (Supplementary Table S1). Among the 796 isolates, the overall percentage of resistance to linezolid was 62.8% (Fig. 1d), with a similar resistance rate of 67.3% observed for enterococci (Fig. 1a). The resistance rates for lactococci and vagococci were relatively lower at 28.8% and 23.8%, respectively (Fig. 1b, c). However, it is worth noting that 59.3% of lactococci exhibited decreased susceptibility to linezolid, leaving only 11.9% of them remained susceptible to this antibiotic. About half of the strains showed resistance to ciprofloxacin, but the rates were relatively higher for lactococci and vagococci, reaching 86.4% and 76.2%, respectively. All vagococci remained susceptible to penicillin G, and this biotic also showed high efficiency against 78.6% of the enterococci obtained. Notably, with the exception of intrinsically resistant strains, no vancomycin-resistant isolates were observed in this study.

### Identification of ARG and OptrA variants

Whole-genome sequencing (WGS) analysis was conducted to comprehensively characterize the genetic features of the selected cohort, comprising 208 enterococci and 86 nonenterococcal isolates. The detailed analysis revealed the presence of five *poxtA*-positive *E. hulanensis* strains (D5-2, D6-4, D19-2, D36-2, and D86-2), one *E. faecalis* strain (M6), and one *E. raffinosus* strain (B646-2) harboring the *poxtA2* variant, which was recently reported in the literature [25]. Furthermore, a total of 36 ARGs were characterized (Supplementary Table S1). These genes collectively confer resistance to phenicols, macrolides, fosfomycin, tetracyclines, trimethoprim, and aminoglycosides, thus aligning with the multiple drug resistance phenotypes previously observed for these isolates.

**Fig. 2:**
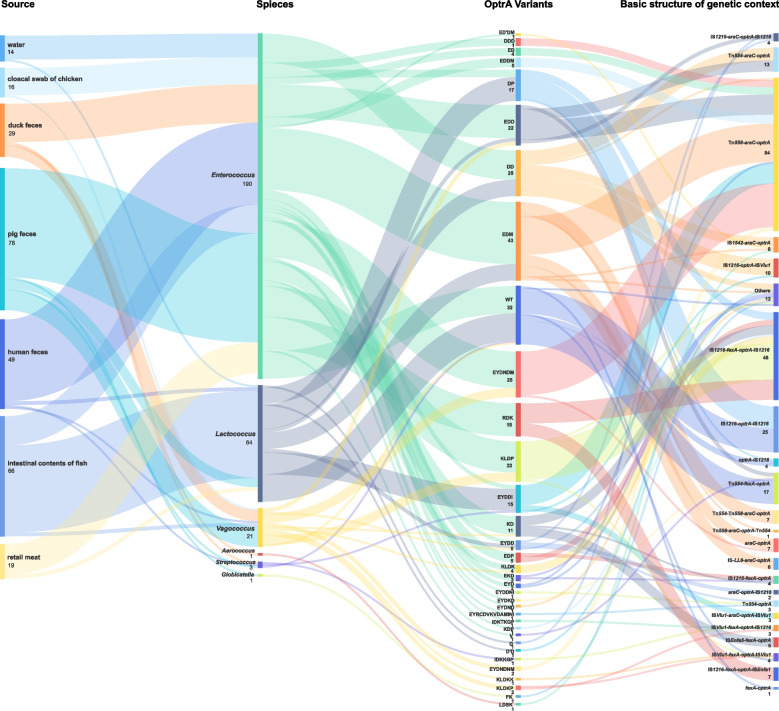
The flow of the source, species, OptrA variants, and basic structure of genetic context of *optrA*-carrying isolates

The alignment of the amino sequence of OptrA_E349_, designated as the wild-type (WT), revealed substitutions in 34 positions of the amino acid sequence (Supplementary Figure S1), with Y176D, K3E, and G393D substitutions being commonly identified. These alterations formed a total of 34 OptrA variants among 217 *optrA*-positive isolates subjected to WGS. The most frequently identified variant was the EDM type (*n* = 43), followed by WT (*n* = 32), DD (*n* = 25), and EYDNDM (*n* = 25). A total of nine novel OptrA variants were designated as ED'DM, EYDDNI, EYDNDNM, EYRCDVKVDAMINI, FK, IDKTKGP, KLDKK, KLDKP, and V (Fig. 2). Corresponding MIC values for each OptrA variant fell within different ranges (Supplementary Figure S1). Higher linezolid MIC values were commonly associated with the WT, EDP, KD, KLDP, and RDK variants, regardless of the genera or species. No distinct genus boundary for the OptrA variants was found, as isolates from different species or sources shared the same or similar OptrA variants. Moreover, in nine strains, binary carriage of two types of OptrA variants within the same strain was observed, including two *E. faecalis* strains (M88 and P42-1), six *L. lactis* strains (M55, Y75, Y7, Y39, Y73, and Y74-2), and one *E. avium* strain (M44-2) (Supplementary Table S1).

### Phylogenetic analysis of linezolid resistance gene-carrying isolates

**Fig. 3:**
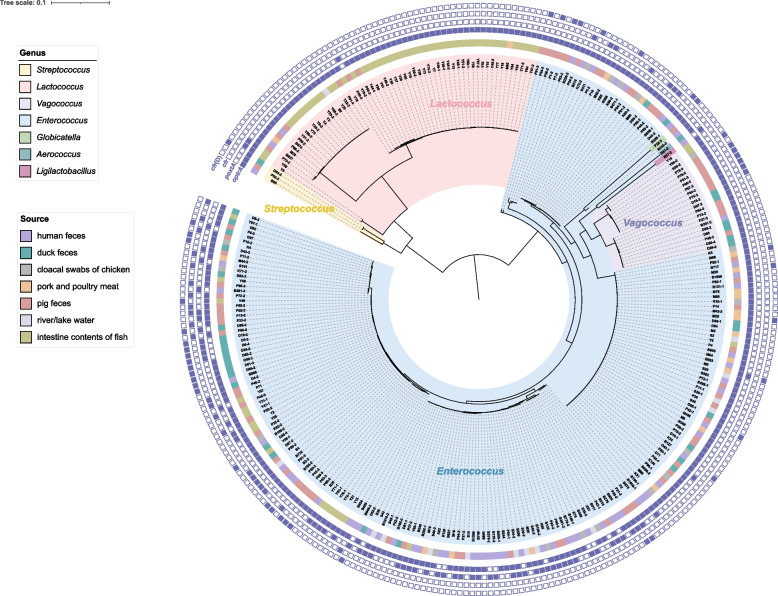
Phylogeny of strains carrying linezolid resistance genes. The phylogenetic tree was generated based on the 16S rRNA sequences of linezolid resistance gene reservoirs by the maximum likelihood method. The genera and source of each strain were illustrated as the legend indicated. The tiny squares with linear arrangements from the inside to the outside indicated the carriage of linezolid resistance genes

To investigate the phylogeny of linezolid resistance reservoirs, multiple alignments of the 16S rRNA sequences of these strains were performed to construct the maximum likelihood tree (Fig. 3). The analysis revealed that the 294 strains from seven genera clustered in different branches, and even strains identified as the same genus displayed significant phylogenetic distance. Moreover, no apparent aggregation of strains from a single source or with the same linezolid resistance gene patterns was observed.

To further investigate the genetic backgrounds and relatedness of linezolid resistance gene-carrying isolates among different niches, SNPs for frequently isolated species were analyzed and mapped. A total of 13 SNP-based maximum-likelihood phylogenetic trees were generated for *Enterococcus* [*E. faecalis* (*n* = 53), *E. faecium* (*n* = 36), *E. avium* (*n* = 34), *E. dongliensis* (*n* = 15), *E. gallinarum* (*n* = 13), *E. casseliflavus* (*n* = 9), *E. raffinosus* (*n* = 9), *E. hirae* (*n* = 7), and *E. hulanensis* (*n* = 6)], *Lactococcus* [*L. lactis* (*n* = 38) and *L. petauri* (*n* = 15)], and *Vagococcus* [*V. lutrae* (*n* = 11) and *V. carniphilus* (*n* = 7)] (Supplementary Figure S2). Generally, large phylogenetic differences were observed for both enterococci and non-enterococci, even strains originating from the same source were dispersed across the different nodes on the tree, indicating the variety of genetic backgrounds of these linezolid resistance reservoirs. However, despite there being obvious clonal aggregation yet to be found, some clades still contained closely related isolates from the same or various sources. A distinct clustering profile of the *optrA*-positive *L. lactis* population in the intestine of fish was mapped (Supplementary Figure S2c), but six strains with different profiles of OptrA variants still showed close relations (Clade A). A similar scenario was also discovered in *V. carniphilus* from pigs (Supplementary Figure S2l). Three isolates of Clade A that displayed one amino acid alteration in OptrA (EYDNDM VS. EYDNDNM) were clonally related with a co-carriage of the *cfr*(D) gene. Similarly, it was of note that the inter-niche clonal spread was always associated with the binary carriage of linezolid resistance genes in our study. Three *E. faecium* isolates derived from water samples of two conjoint rivers were clustered together (Clade A) with the coexisting of the *optrA*_DD_ and *poxtA* genes (Supplementary Figure S2b). For *E. avium*, five highly related isolates from duck feces displayed the co-occurrence of the *optrA*_EDM_ and *cfr*(D) genes (Clade A, Supplementary Figure S2d). The clonal dissemination of binary linezolid resistance gene carriers was also observed in Clade A of *E. raffinosus* from fish samples that were positive for *optrA*_EDDM_ and *poxtA* genes (Supplementary Figure S2j), as well as *E. hulanensis* of duck origin that carried *poxtA2* and *cfr*(D) (Supplementary Figure S2m). Not only the single clonal spread was observed, but two individual clades of *L. petauri* of fish origin were clustered as Clade A and Clade B, the isolates also showed a close genetic distance inner each clade (Supplementary Figure S2f).

The potential epidemiology linkages were also noticed across niches boundary. Two *E. avium* strains of human origin and four strains of porcine origin were clustered closely together on Clade B of the phylogenetic tree (Supplementary Figure S2d). All six of these strains showed the binary carriage of both *optrA*_EDM_ and *cfr*(D) genes. In the case of *E. faecalis*, ST16 was the most frequently identified sequence type, displaying a polyclonal structure in the phylogenetic tree (Supplementary Figure S2a). However, this ST type was still shared by strains from various sources, including human feces (*n* = 2), duck feces (*n* = 3), pig feces (*n* = 1), pork and poultry meat (*n* = 3), and water samples (*n* = 1). Following that, the ST116 (*n* = 4) was also shared by the strains derived from the feces of humans (*n* = 1) and pigs (*n* = 1) as well as water samples (*n* = 2), indicating ST16 and ST116 types of *E. faecalis* may act as the vehicle for the transmission of linezolid resistance among different niches. Despite the linkage for the inter-niche clonal spread was not as evident as that within a single niche, closely related strains from various sources still demonstrated the phylogenomic relationships and potential transmission routes of these linezolid resistance reservoirs.

### Genetic environment of linezolid resistance genes

The arrays for the genetic environment of the *optrA*, *poxtA*, *poxtA2*, *cfr*, and *cfr*(D) genes were represented as illustrations in Supplementary Figure S3. A high variation of *optrA*-carrying genetic platforms with abundance and diversity in MGEs and ARGs was observed, which yielded plenty of different arrangements of genetic environment. IS*1216E* (*n* = 41) was mostly identified upstream and/or downstream of the *optrA* gene as previous studies extensively described [9]. Further, one or multiple copies of assorted other ISs including IS*Vlu1* (*n* = 17), IS*Enfa1* (*n* = 5), IS*Enfa5* (*n* = 4), IS*-LL6* (*n* = 3), IS*256* (*n* = 1), IS*1251* (*n* = 1), and IS*S1N* (*n* = 1) were also found to flank the *optrA*-core regions. Transposons were found in the vicinity of *optrA* as well, including the complete Tn*554* and Tn*558* or their relics in different sizes. However, the sequence at the junctions of the transposons varied with respect to the target sites, indicating the different origin and integration routes by individual mobilization events. The co-location of other ARGs of these *optrA*-carrying contigs was characterized, with *fexA* (*n* = 62) and *erm*(A) (*n* = 53) being most commonly detected, further contributing to the co-selection of *optrA*.

To better clarify the corresponding among these genetic environments with the OptrA variants, species of hosts, and sources, the 105 arrangements were simplified to 22 basic types as Fig. 2 listed. The arrangement of Tn*558*-*araC*-*optrA* (*n* = 84) was most prevalent among the *optrA*-positive isolates. Following that, the arrangements of IS*1216*-*fexA*-*optrA*-IS*1216* (*n* = 48), and IS*1216*- *optrA*-IS*1216* (*n* = 25) were also common. The same or similar arrangements were shared by different *optrA* variants, suggesting the possible interconnection of these strains and a lack of conservation of *optrA* during dissemination.

The genetic environment for the *poxtA* gene was relatively consistent (Supplementary Figure S3j), while IS*1216E* was found to flank these contigs in the same or opposite orientations. The recently reported *poxtA2* gene, whose 3′ end was not disrupted by IS*1216E* but directly associated with the *cfr *(D) was discovered in seven enterococci of different species and sources, and the arrangement of these contigs was homologous to that identified in porcine manure [25]. Consistent with that reported elsewhere, the *cfr*(D) gene was commonly associated with a complete or truncated *guaA* gene downstream and a truncated IS*Seq2* upstream, flanked or not by IS*1216E* (Supplementary Figure S3j). However, it was of note that the insertion of *optrA*_WT_ into the common arrangement of *cfr*(D) thus generated the array of IS*1216E*-ΔIS*Seq2*-*cfr*(D)-*optrA*-IS*1216E* (Supplementary Figure S3h). It was observed in *S. parauberis* D84-2, an unreported reservoir for these two linezolid resistance genes. The IS*Enfa5*-*cfr*-IS*Enfa5* genetic array was most frequently identified in the *cfr*-carrying enterococci (*n* = 16), followed by that of IS*256*-*cfr*-IS*256* (*n* = 6), both of which were dissimilar with that characterized in staphylococci in our earlier study [30]. Overall, though showed diversity, these genetic contexts of linezolid resistance genes were shared by the heterogeneous reservoirs in the levels of species and sources, indicating the horizontal transfer and potential interconnections of these reservoirs.

## Discussion

Representative studies on surveillance of the epidemiological situation of linezolid resistance via the One Health approach in various niches have been conducted on a global scale. However, comprehensive and multifield research on the resistance to this last-line resort antibiotic is still rare, and its prevalence has been underestimated. This might be attributed to the urgent need to optimize sensitive and standardized methodologies for the detection of ARGs. Our previous study reported a human intestinal carriage rate of *optrA* at 19.3% with an optimized high-sensitivity screening approach, which displayed a more than tripled rate compared to the traditional method currently widely used [52]. We used this method to screen for samples from multiple niches to comprehensively perceive the epidemiologic phenomenon of all linezolid resistance genes, beyond only focusing on clinical settings. As a result, we discovered an unexpectedly high prevalence and a large diversity of linezolid resistance reservoirs at multiple levels.

The wide distribution of linezolid resistance genes across various ecological niches has been emphasized, and the remarkably high prevalence of the *optrA* gene has raised significant concern. In addition, our study provided comprehensive knowledge on the prevalence of large-scale samples of the *cfr*(D) gene, which was also relatively high among these resistance genes. The animal-origin samples were identified as important reservoirs for linezolid resistance in our study, suggesting that these resistance determinants might have accumulated in the intestinal microbiota of animals. Despite linezolid not being approved for use in the livestock or poultry industries, our findings demonstrated that the distribution of linezolid resistance genes could be affected by the residual concentration of florfenicol in livestock manures [64]. This indicates that the extensive use of veterinary antibiotics may lead to the enrichment and dissemination of acquired linezolid resistance genes in the gut microbiota of livestock through co-selection. The high prevalence of linezolid resistance genes among farm animals is a cause for concern, as these resistance determinants may enter the food chain via various routes, such as spillover at the slaughterhouse or contamination of the circulated water as a form of excretion [65]. This could potentially result in human intestinal colonization and therapeutic failure, which poses a potential risk to public health [66].

Our study also unveiled the remarkable diversity in linezolid resistance, exemplified by the heterogeneous backgrounds of the resistance reservoirs. Firstly, we made a valuable addition to the database of host species carrying linezolid resistance genes. Though enterococci showed a predominance in harboring the four linezolid resistance genes, we revealed the previously neglected role of multiple non-enterococcal species, indicating the widespread distribution of these genes in Gram-positive communities. Notably, the previously unnoticed *L. lactis* emerged as the most abundant species carriering the *optrA* gene in the intestinal of fish. Additionally, *optrA* and *poxtA* were observed in other *Lactococcus* spp., suggesting the relevance of linezolid resistance dissemination in the ecosystem for these bacteria as commensal intestinal flora of humans and animals [67]. The *Vagococcus* spp. also behaved as an important linezolid reservoir, which was equally represented in a recent study in Europe [44]. The occurrence of linezolid resistance genes in less frequently studied species indicates that the reservoirs we have currently discovered likely represent only a fraction of the larger picture, and many uncharacterized individuals with widespread phenomena remain to be identified.

Even within *Enterococcus* spp., which is considered one of the best-studied Gram-positive genera, we observed a diverse array of species carrying linezolid resistance genes, including less identified enterococci such as *E. hulanensis*, *E. viikkiensis*, and *E. xiangfangensi*s, among others. The carriage of linezolid resistance genes in these infrequent enterococci is noteworthy, particularly considering that enterococci have been proposed for monitoring antibiotic resistance in food animals [65]. These less-common enterococci are easily overlooked during surveillance, making their identification crucial for comprehensive monitoring efforts. Further, not only did we observe species diversity, but strains of the same species displayed substantial phylogenetic distances or possessed different linezolid resistance genes. The polyclonal structure of each species population further underscores the highly diverse phylogenetic lineages of the embedded linezolid resistance genes, consistent with findings from previous studies [34, 41, 68].

Another dimension of diversity in linezolid resistance was manifested in the highly variable genetic platforms of these resistance genes. We discovered a wealth of MGEs in the flanking regions of linezolid resistance genes, potentially playing a crucial role in the horizontal transfer of these resistance determinants. Among those elements, the significance of IS*1216* was emphasized, as it was most frequently identified and has been shown to facilitate the mobilization of *optrA*- and *poxtA*- carrying contigs in the form of IS*1216*-based translocatable units [23, 26]. Furthermore, these linezolid resistance loci were also commonly associated with the co-selection of multiple additional ARGs, potentially promoting the persistence of these linezolid resistance genes within the Gram-positive population. The abundance and diversity of MGEs and ARGs are indicative of genetic structural plasticity, which is also evident within the linezolid resistance genes themselves, as reflected in the presence of multiple gene variants.

The greatest genetic flexibility was observed in the highly variable amino acid sequence of OptrA, a distinctive feature of the *optrA* gene contributing to the diverse linezolid resistance levels we observed. However, the carriage of OptrA profiles did not consistently correspond to specific resistance phenotypes, suggesting the involvement of potential additional mechanisms.

Despite the extensive diversity observed, there were still certain similarities and potential relatedness between these linezolid resistance reservoirs. Shared gene variants and genetic contexts among heterogeneous reservoirs of linezolid resistance, underscored the flow of linezolid resistance genes in the ecosystem. The absence of represented epidemic clones we observed suggested that these linezolid resistance determinants could be readily acquired or exchanged through horizontal transfer, facilitated by the abundance of various insertion sequences and transposons that enable mobilization and transposition events. Clonal spread also contributed to the dissemination of linezolid resistance, primarily within specific niches. Although its significance was less pronounced across diverse niches, some strains of different origins still displayed limited phylogenetic distance, implying potential epidemiological linkages among multiple niches.

One of the limitations of this study pertains to the sample collection, which was conducted in a single-center manner, and as such, the epidemic features observed only represent the situation in Hangzhou City. To gain a more comprehensive understanding of the circulation of these linezolid resistance genes, further work and investigations on a larger geographical scale are warranted. As *optrA*, *poxtA*, *cfr*, and *cfr*-like genes confer a wide range of MIC values to linezolid (varied from 2 to 32 mg/L) while higher values to florfenicol at least for 16 mg/L; thus, we chose florfenicol to isolate the target isolates. The unexpectedly high prevalence reflected by this study methodology may showed slight discrepancy but was much closer to the true prevalence.

## Conclusions

In conclusion, our study has provided comprehensive knowledge of linezolid resistance at the human-animal-environment interface and highlighted a remarkably high prevalence of the *optrA* gene in multiple niches. Additionally, we have reported the previously unexplored prevalence of *poxtA*, *cfr*, and *cfr*(D) genes, collectively demonstrating that animal intestines and associated settings serve as important linezolid resistance gene reservoirs. These resistance determinants have embedded into a wide range of genetic backgrounds and represent a prevalent situation among Gram-positive communities, not only in enterococci as the predominant host, but also in a variety of non-enterococcal species, including *Lactococcus*, *Vagococcus*, *Streptococcus*, *Aerococcus*, and *Globicatella*. The horizontal transfer facilitated by MGEs in the flanking region of linezolid resistance genes has been identified as the primary mechanism responsible for the wide dissemination of linezolid resistance on a large scale. Additionally, the clonal spread has also contributed to the spread of resistance. Our study provides evidence of potential clonal relatedness and epidemiology linkage among heterogenous niches, it is important to note that further research is needed to fully understand the circulation of linezolid resistance genes in the ecosystem. Nevertheless, the data obtained in this study can serve as a valuable baseline for assessing the capacity of linezolid resistance genes in multiple niches, demonstrating the power of One Health surveillance in informing policies aimed at improving public health.

### Supplementary Information

**Additional file 1: Supplementary Table S1.** Characteristics of strains carrying linezolid resistance genes from multiple niches. Detailed information of linezolid resistance reservoirs isolated in this study regarding their source, species, antimicrobial susceptibility profiles, carriage of antimicrobial resistance genes, and genetic context of linezolid resistance genes were illustrated.

**Additional file 2: Supplementary Figure S1.** Representation of alignment of the amino acid sequence and relevant linezolid MIC values for OptrA variants. Substitutions (green square) in amino sequence, when compared to the wild-type OptrA sequence (yellow square), were illustrated as indicated. The numbers in the blue square represented the numbers for strains that displayed the corresponding linezolid MICs of certain species, while the grey ones represented no corresponding strains in our study.

**Additional file 3: Supplementary Figure S2.** Phylogenetic maximum-likelihood tree of linezolid resistance reservoirs. The SNP-based phylogenetic tree for *E. faecalis *(a), *E. faecium *(b),* L. lactis *(c), *E. avium *(d), *E. dongliensis* (e), *L. petauri *(f), *E. gallinarum *(g), *V. lutrae *(h), *E. casseliflavus* (i), *E. raffinosus* (j), *E. hirae* (k), *V. carniphilus *(l), and *E. hulanensis *(m). The sample type, source, and OptrA variants were illustrated as the legend indicated. The * represented the two types of OptrA variants in the strain as indicated. The tiny squares with linear arrangements from the inside to the outside indicated the carriage of antimicrobial resistance genes as the label indicated. The filled squares represented the presence of this gene in the genome of this isolate and vice versa. The strains closely related were labeled in the yellow or green shade.

**Additional file 4: Supplementary Figure S3.** The genetic environment of the linezolid resistance genes. The arrows indicate the positions and directions of transcription of the different genes. All insertion sequences were labeled as orange, antimicrobial resistance genes were labeled as red, gene encoding proteins with known functions were labeled as blue, the *rep* genes were labeled as green, and the hypothesis proteins were labeled as grey. The letters at the lower right corner of *optrA* indicated the OptrA variants. N represented the number of strains that harbored the corresponding resistance locus encoding the gene or variants. Δ symbol indicates a truncated gene.

## Data Availability

The whole-genome sequencing data set was retrieved with BioProject accession numbers PRJNA948548 and PRJNA848114.

## Abbreviations

AMR: Antimicrobial resistance
MRSA: Methicillin-resistant *Staphylococcus aureus*
VRE: Vancomycin-resistant enterococci
ABC: ATP-binding cassette
MGE: Mobile genetic element
ANI: Average Nucleotide Identity
MIC: Minimal inhibitory concentrations
CLSI: Clinical and Laboratory Standards Institute
WGS: Whole-genome sequencing
ARG: Antimicrobial resistance gene
ST: Sequence type
SNP: Single-nucleotide polymorphism
WT: Wild-type
