# Supplementary material for: Mapping the widespread distribution and transmission dynamics of linezolid resistance in humans, animals, and the environment
Source: Microbiome. 2024 Mar 13;12:52. doi: 10.1186/s40168-023-01744-2 (PMC10936028; doi:10.1186/s40168-023-01744-2)
Supplement: Supplementary file 4 — Additional file 3: Supplementary Figure S2. Phylogenetic maximum-likelihood tree of linezolid resistance reservoirs. The SNP-based phylogenetic tree for E. faecalis (a), E. faecium (b), L. lactis (c), E. avium (d), E. dongliensis (e), L. petauri (f), E. gallinarum (g), V. lutrae (h), E. casseliflavus (i), E. raffinosus (j), E. hirae (k), V. carniphilus (l), and E. hulanensis (m). The sample type, source, and OptrA variants were illustrated as the legend indicated. The * represented the two types of OptrA variants in the strain as indicated. The tiny squares with linear arrangements from the inside to the outside indicated the carriage of antimicrobial resistance genes as the label indicated. The filled squares represented the presence of this gene in the genome of this isolate and vice versa. The strains closely related were labeled in the yellow or green shade. [file 40168_2023_1744_MOESM3_ESM.pdf]

Figure S2

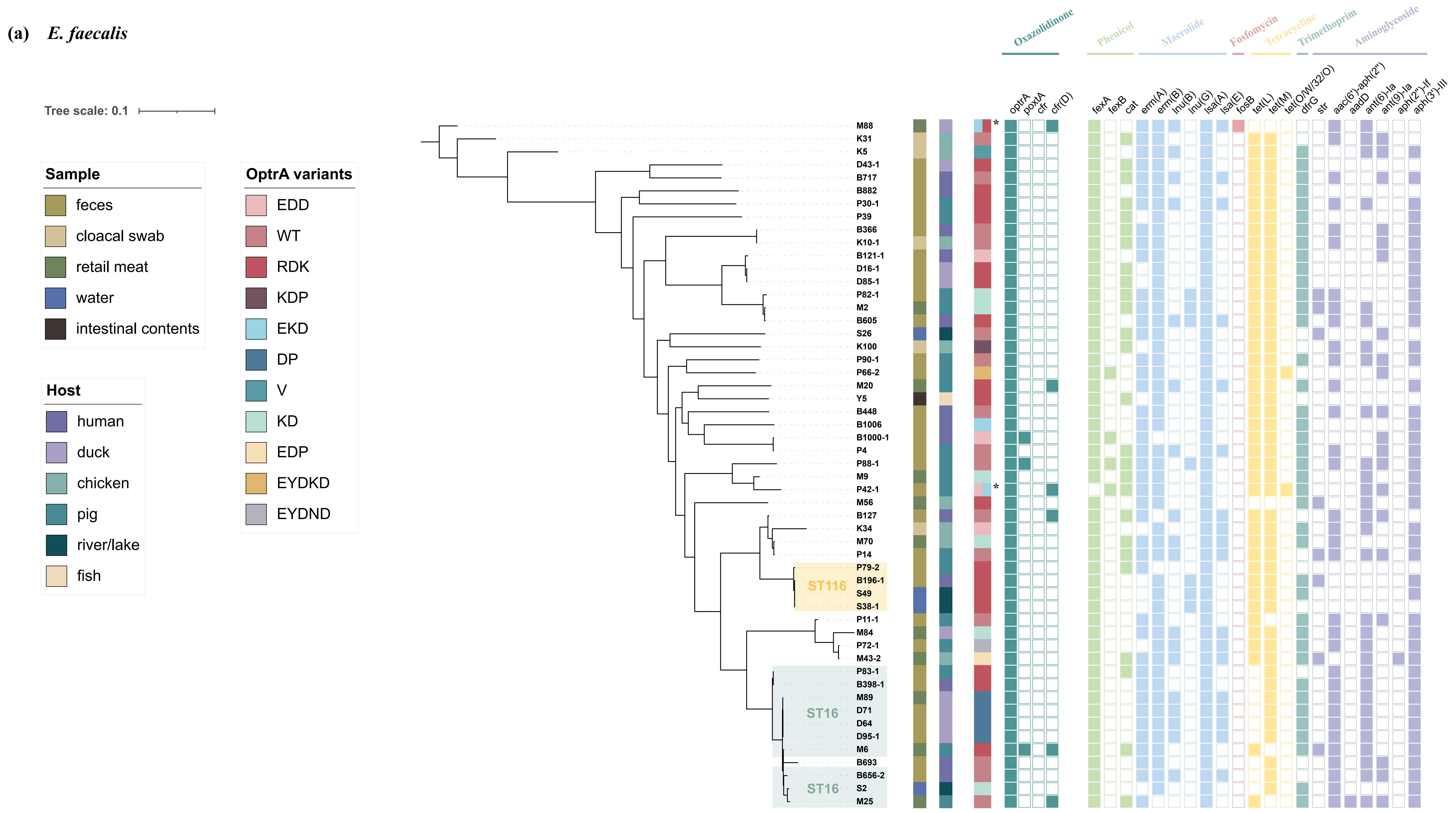
(a) E. faecalis
Tree scale: 0.1
Sample
feces
cloacal swab
retail meat
water
intestinal contents
OptrA variants
EDD
WT
RDK
KDP
EKD
DP
V
KD
EDP
EYDKD
EYDND
Host
human
duck
chicken
pig
river/lake
fish
Oxazolidinone
Phenicol
Macrolide
Fosfomycin
Tetracycline
Trimethoprim
Aminoglycoside
optrA
poxtA
cfr
cfr(D)
fexA
fexB
cat
erm(A)
erm(B)
lnu(B)
lnu(G)
lsa(A)
lsa(E)
fosB
tet(L)
tet(M)
tet(O/W/32/O)
dfrG
str
aac(6')-aph(2")
aadD
ant(6)-Ia
ant(9)-Ia
aph(2")-If
aph(3')-III
M88
K31
K5
D43-1
B717
B882
P30-1
P39
B366
K10-1
B121-1
D16-1
D85-1
P82-1
M2
B605
S26
K100
P90-1
P66-2
M20
Y5
B448
B1006
B1000-1
P4
P88-1
M9
P42-1
M56
B127
K34
M70
P14
P79-2
B196-1
S49
S38-1
P11-1
M84
P72-1
M43-2
P83-1
B398-1
M89
D71
D64
D95-1
M6
B693
B656-2
S2
M25
ST116
ST16
ST16

Figure S2

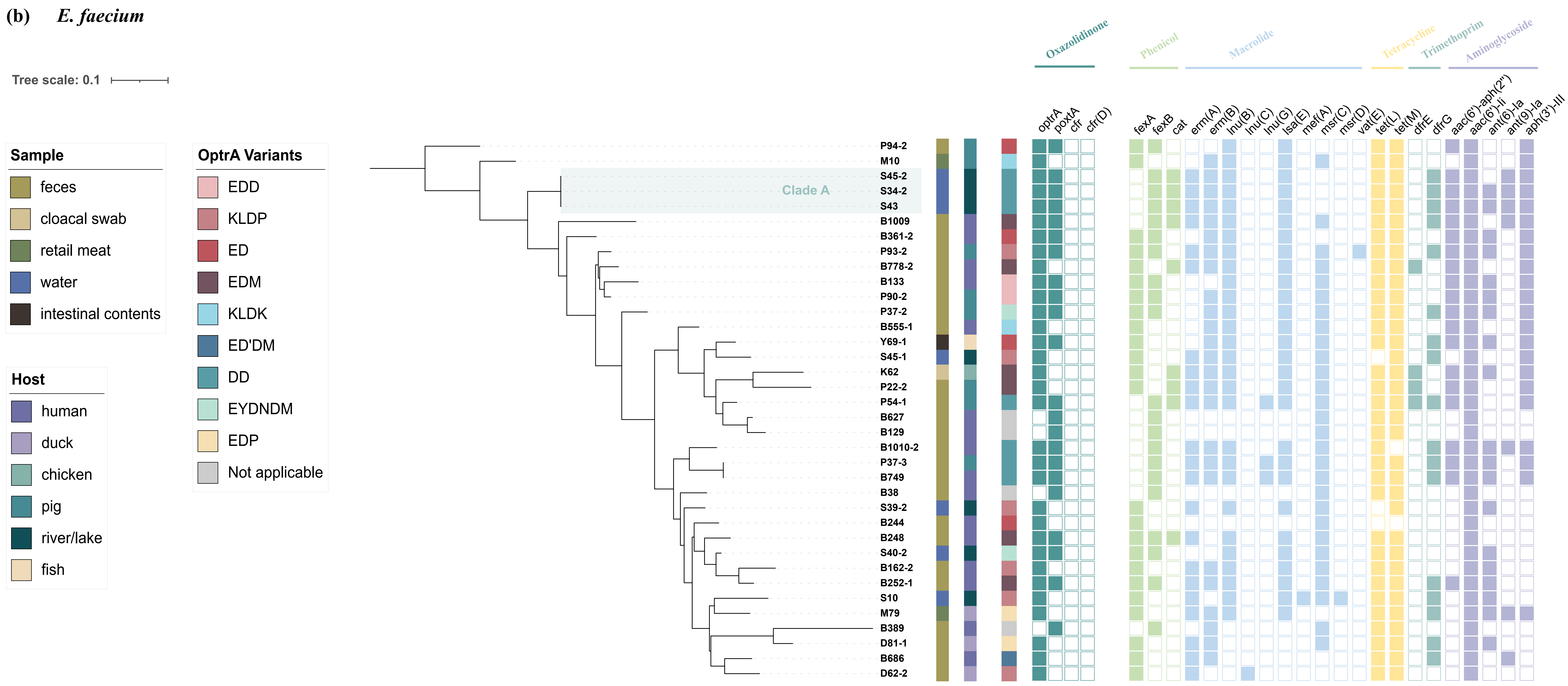

(b) E. faecium
Tree scale: 0.1
Sample
feces
cloacal swab
retail meat
water
intestinal contents
OptrA Variants
EDD
KLDP
ED
EDM
KLDK
ED'DM
DD
EYDNDM
EDP
Not applicable
Host
human
duck
chicken
pig
river/lake
fish
Clade A
Oxazolidinone
Phenicol
Macrolide
Tetracycline
Trimethoprim
Aminoglycoside
optrA
poxtA
cfr
cfr(D)
fexA
fexB
cat
erm(A)
erm(B)
lnu(B)
lnu(C)
lnu(G)
lsa(E)
mef(A)
msr(C)
msr(D)
vat(E)
tet(L)
tet(M)
dfrE
dfrG
aac(6')-aph(2")
aac(6')-Ii
ant(6)-Ia
ant(9)-Ia
aph(3')-III
P94-2
M10
S45-2
S34-2
S43
B1009
B361-2
P93-2
B778-2
B133
P90-2
P37-2
B555-1
Y69-1
S45-1
K62
P22-2
P54-1
B627
B129
B1010-2
P37-3
B749
B38
S39-2
B244
B248
S40-2
B162-2
B252-1
S10
M79
B389
D81-1
B686
D62-2

Figure S2

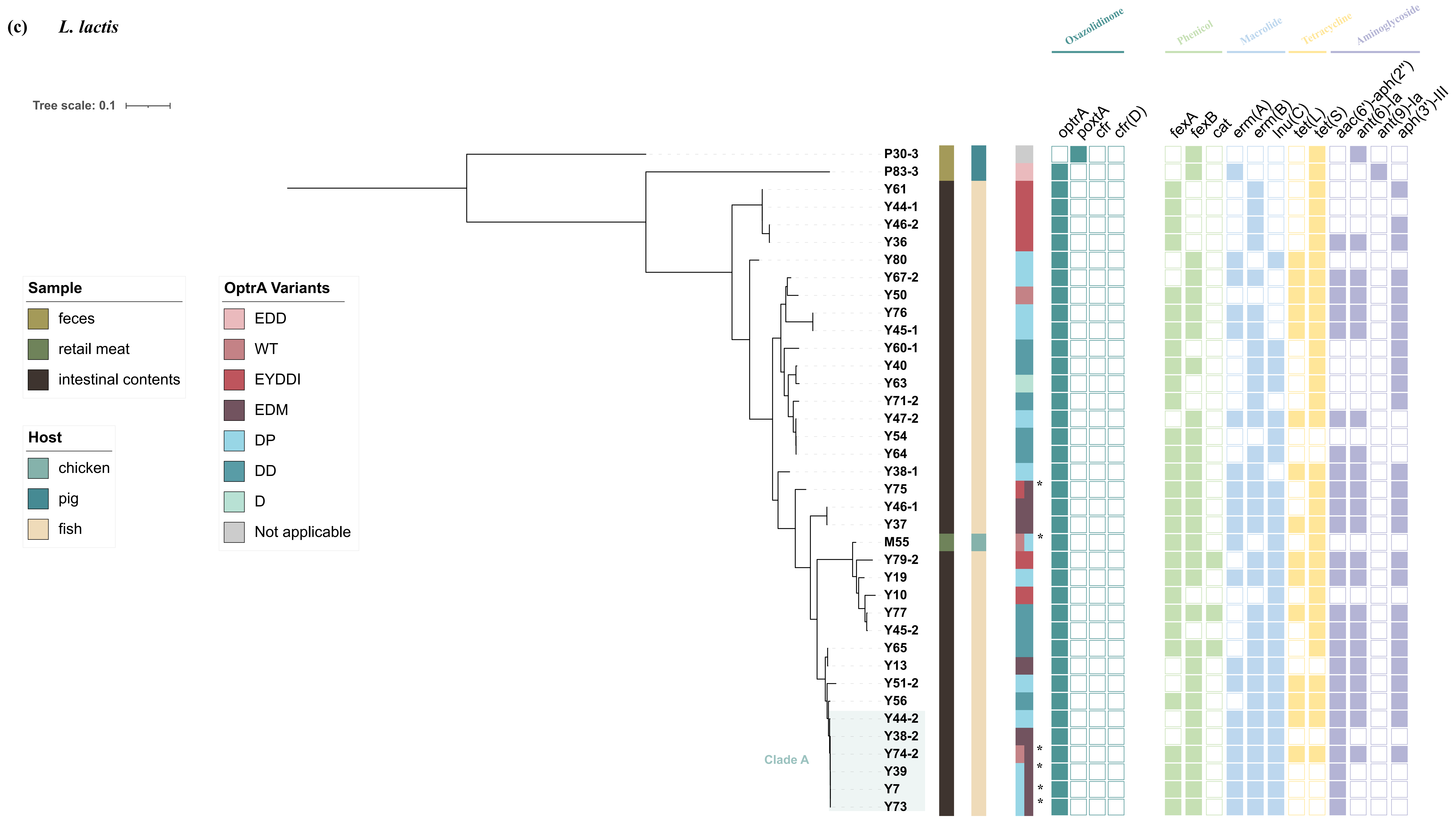

(c)
L. lactis
Tree scale: 0.1
Oxazolidinone
Phenicol
Macrolide
Tetracycline
Aminoglycoside
optrA
poxtA
cfr
cfr(D)
fexA
fexB
cat
erm(A)
erm(B)
lnu(C)
tet(L)
tet(S)
aac(6')-aph(2'')
ant(6)-Ia
ant(9)-Ia
aph(3')-III
P30-3
P83-3
Y61
Y44-1
Y46-2
Y36
Y80
Y67-2
Y50
Y76
Y45-1
Y60-1
Y40
Y63
Y71-2
Y47-2
Y54
Y64
Y38-1
Y75
Y46-1
Y37
M55
Y79-2
Y19
Y10
Y77
Y45-2
Y65
Y13
Y51-2
Y56
Y44-2
Y38-2
Y74-2
Y39
Y7
Y73
Clade A
Sample
feces
retail meat
intestinal contents
OptrA Variants
EDD
WT
EYDDI
EDM
DP
DD
D
Not applicable
Host
chicken
pig
fish

Figure S2

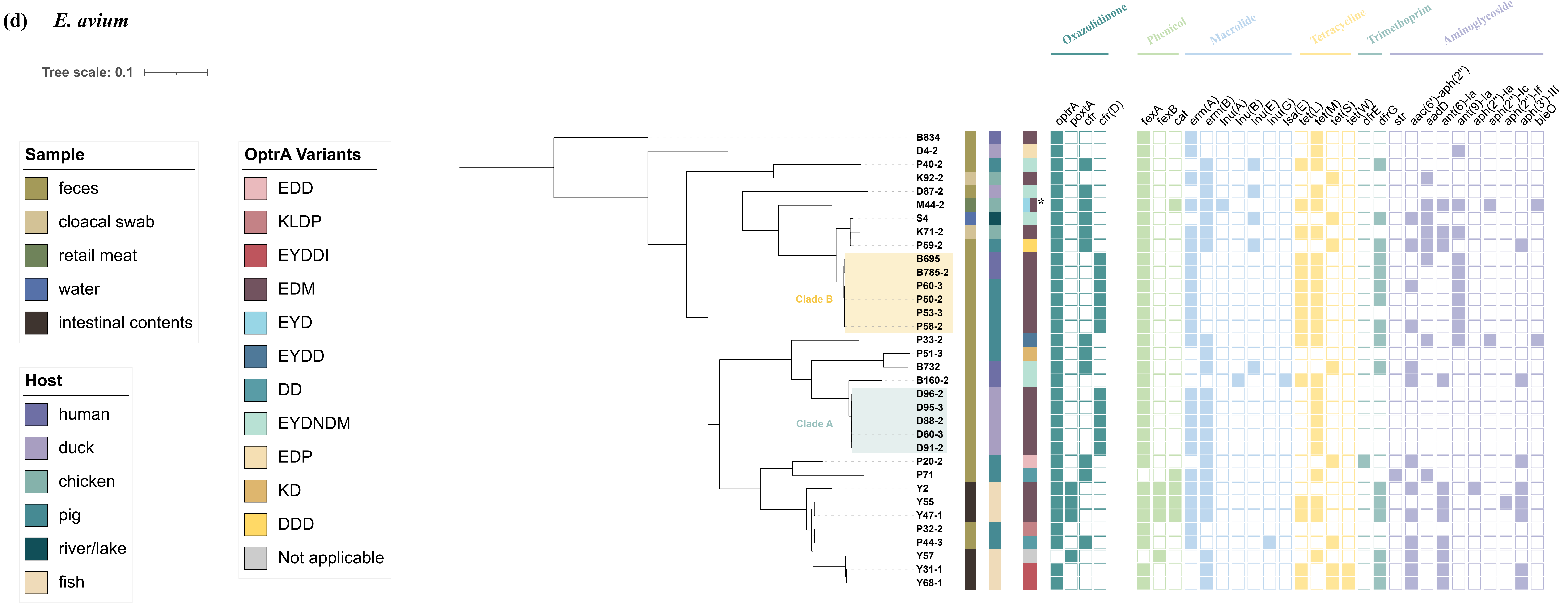
(d) E. avium
Tree scale: 0.1
Sample
feces
cloacal swab
retail meat
water
intestinal contents
Host
human
duck
chicken
pig
river/lake
fish
OptrA Variants
EDD
KLDP
EYDDI
EDM
EYD
EYDD
DD
EYDNDM
EDP
KD
DDD
Not applicable
Oxazolidinone
Phenicol
Macrolide
Tetracycline
Trimethoprim
Aminoglycoside
optrA
poxtA
cfr
cfr(D)
fexA
fexB
cat
erm(A)
erm(B)
lnu(A)
lnu(B)
lnu(E)
lnu(G)
lsa(E)
tet(L)
tet(M)
tet(S)
tet(W)
dfrE
dfrG
str
aac(6')-aph(2'')
aadD
ant(6)-Ia
ant(9)-Ia
aph(2'')-Ia
aph(2'')-Ic
aph(2'')-If
aph(3')-III
bleO
Clade B
Clade A
B834
D4-2
P40-2
K92-2
D87-2
M44-2
S4
K71-2
P59-2
B695
B785-2
P60-3
P50-2
P53-3
P58-2
P33-2
P51-3
B732
B160-2
D96-2
D95-3
D88-2
D60-3
D91-2
P20-2
P71
Y2
Y55
Y47-1
P32-2
P44-3
Y57
Y31-1
Y68-1

Figure S2

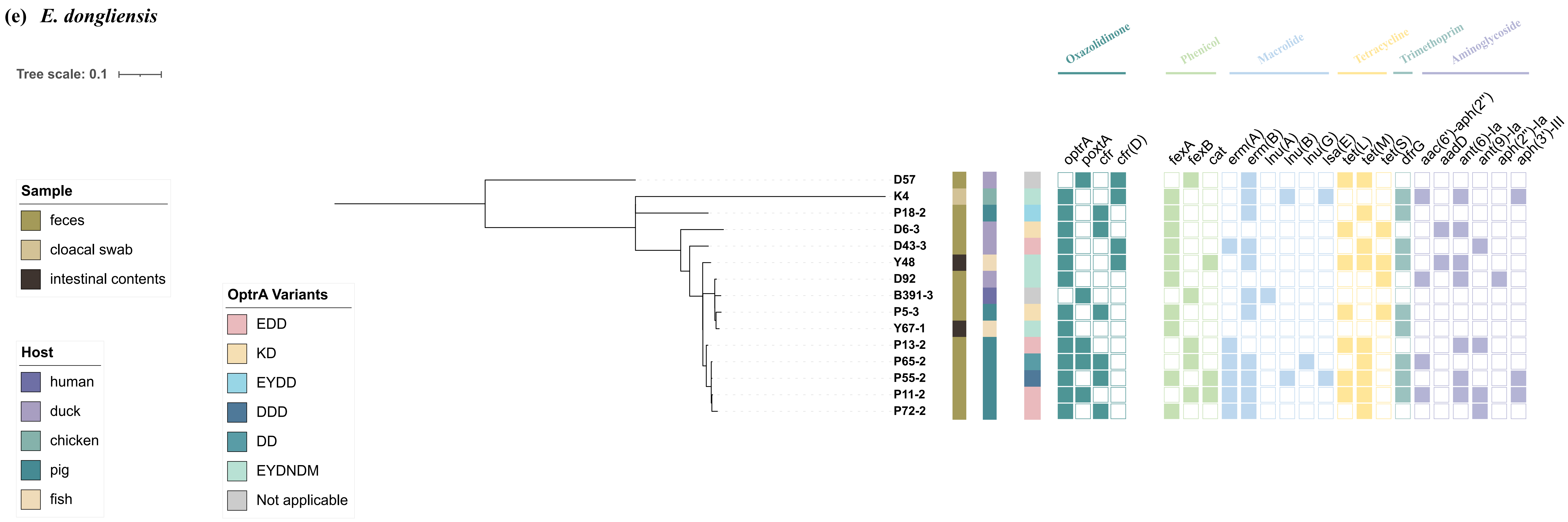
(e) E. dongliensis
Tree scale: 0.1
Oxazolidinone
Phenicol
Macrolide
Tetracycline
Trimethoprim
Aminoglycoside
optrA
poxtA
cfr
cfr(D)
fexA
fexB
cat
erm(A)
erm(B)
lnu(A)
lnu(B)
lnu(G)
lsa(E)
tet(L)
tet(M)
tet(S)
dfrG
aac(6')-aph(2")
aadD
ant(6)-Ia
ant(9)-Ia
aph(2")-Ia
aph(3')-III
D57
K4
P18-2
D6-3
D43-3
Y48
D92
B391-3
P5-3
Y67-1
P13-2
P65-2
P55-2
P11-2
P72-2
Sample
feces
cloacal swab
intestinal contents
Host
human
duck
chicken
pig
fish
OptrA Variants
EDD
KD
EYDD
DDD
DD
EYDNDM
Not applicable

Figure S2

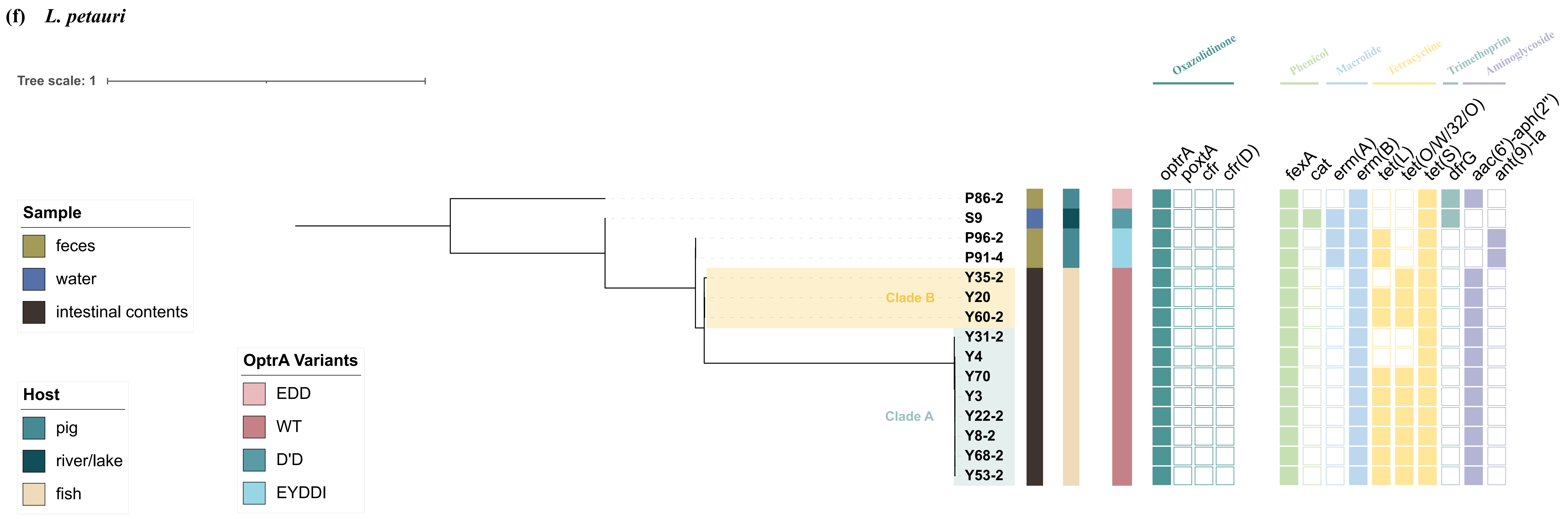

(f) L. petauri
Tree scale: 1
Sample
feces
water
intestinal contents
Host
pig
river/lake
fish
OptrA Variants
EDD
WT
D'D
EYDDI
Clade B
Clade A
P86-2
S9
P96-2
P91-4
Y35-2
Y20
Y60-2
Y31-2
Y4
Y70
Y3
Y22-2
Y8-2
Y68-2
Y53-2
Oxazolidinone
Phenicol
Macrolide
Tetracycline
Trimethoprim
Aminoglycoside
optrA
poxtA
cfr
cfr(D)
fexA
cat
erm(A)
erm(B)
tet(L)
tet(O/W/32/O)
tet(S)
dfrG
aac(6')-aph(2'')
ant(9)-Ia

Figure S2

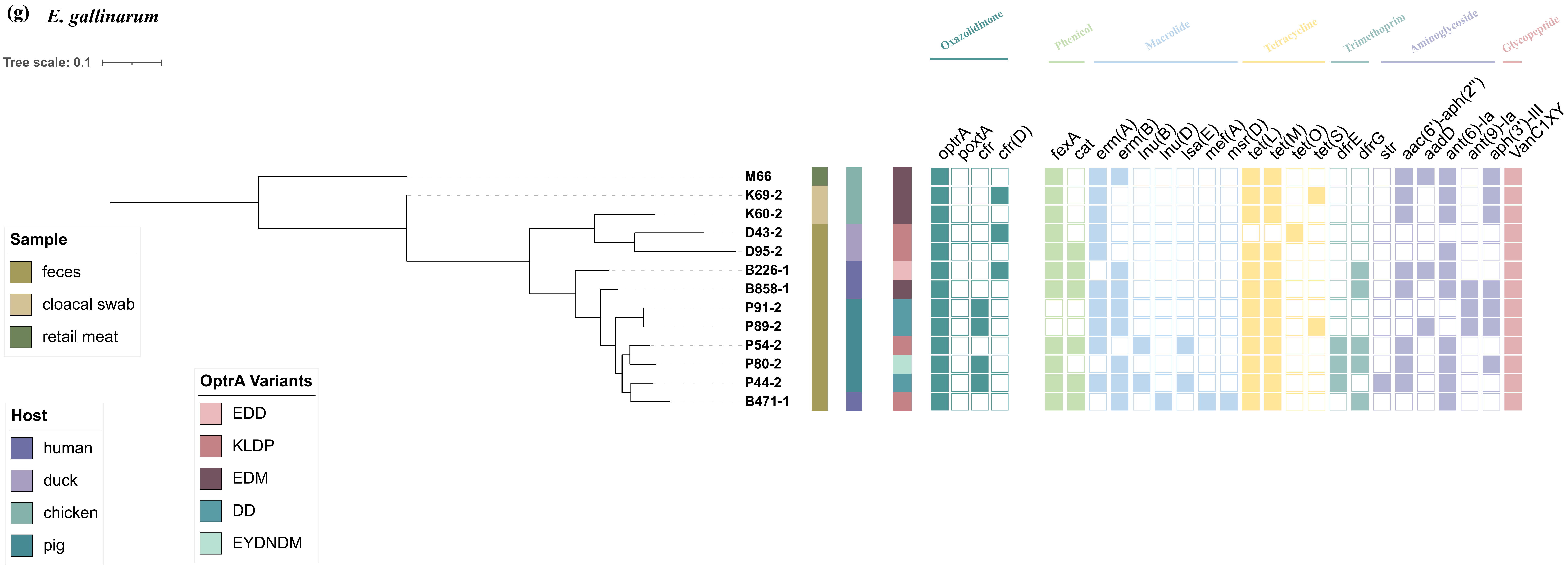
(g) E. gallinarum
Tree scale: 0.1
Oxazolidinone
Phenicol
Macrolide
Tetracycline
Trimethoprim
Aminoglycoside
Glycopeptide
optrA
poxtA
cfr
cfr(D)
fexA
cat
erm(A)
erm(B)
lnu(B)
lnu(D)
lsa(E)
mef(A)
msr(D)
tet(L)
tet(M)
tet(O)
tet(S)
dfrE
dfrG
str
aac(6')-aph(2'')
aadD
ant(6)-Ia
ant(9)-Ia
aph(3')-III
VanC1XY
M66
K69-2
K60-2
D43-2
D95-2
B226-1
B858-1
P91-2
P89-2
P54-2
P80-2
P44-2
B471-1
Sample
feces
cloacal swab
retail meat
Host
human
duck
chicken
pig
OptrA Variants
EDD
KLDP
EDM
DD
EYDNDM

Figure S2

(h) *V. lutrae*

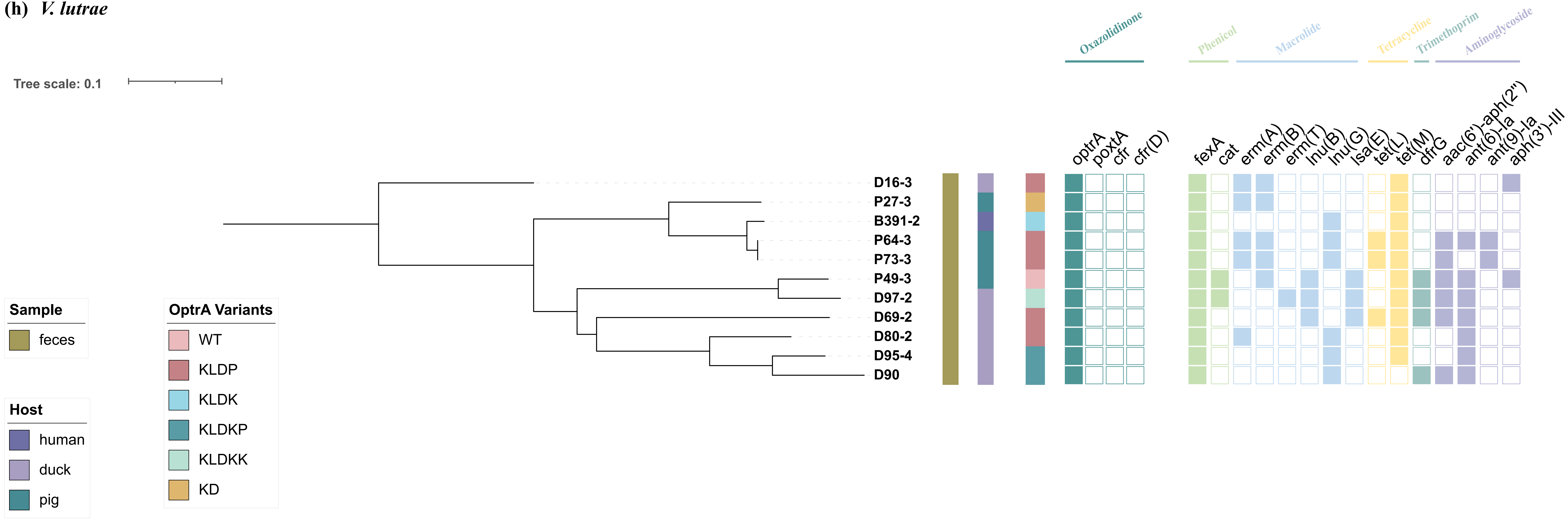

Figure S2

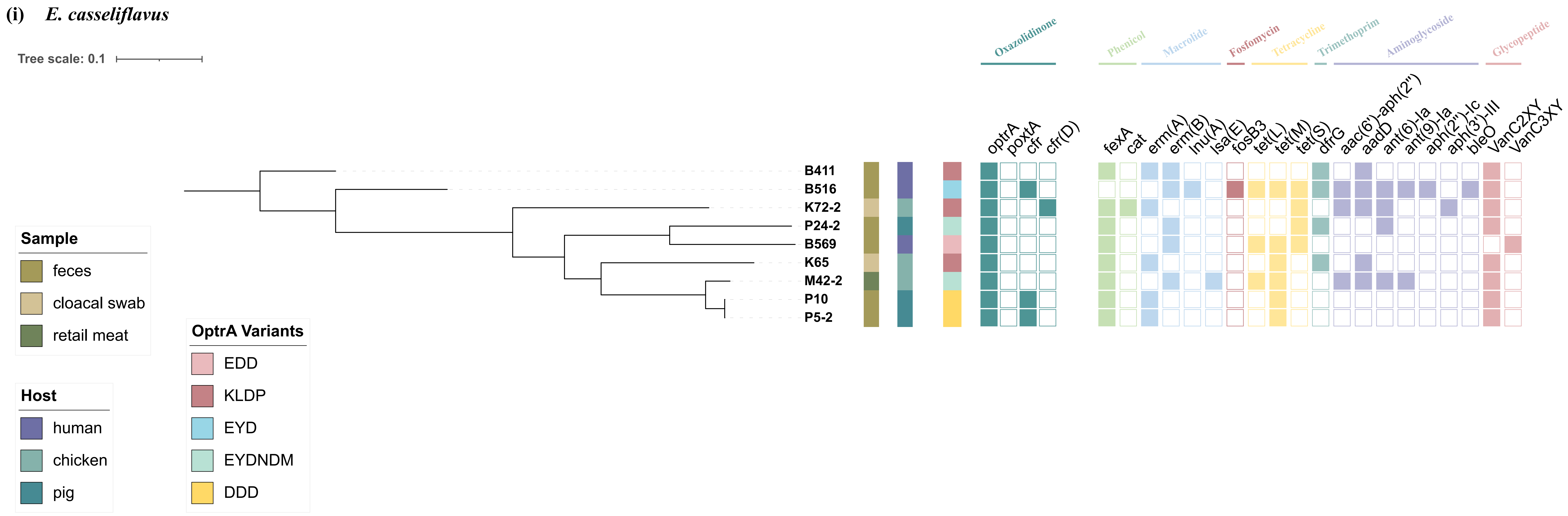
(i) E. casseliflavus
Tree scale: 0.1
Oxazolidinone
Phenicol
Macrolide
Fosfomycin
Tetracycline
Trimethoprim
Aminoglycoside
Glycopeptide
optrA
poxtA
cfr
cfr(D)
fexA
cat
erm(A)
erm(B)
lnu(A)
lsa(E)
fosB3
tet(L)
tet(M)
tet(S)
dfrG
aac(6')-aph(2")
aadD
ant(6)-Ia
ant(9)-Ia
aph(2")-Ic
aph(3')-III
bleO
VanC2XY
VanC3XY
B411
B516
K72-2
P24-2
B569
K65
M42-2
P10
P5-2
Sample
feces
cloacal swab
retail meat
Host
human
chicken
pig
OptrA Variants
EDD
KLDP
EYD
EYDNDM
DDD

Figure S2

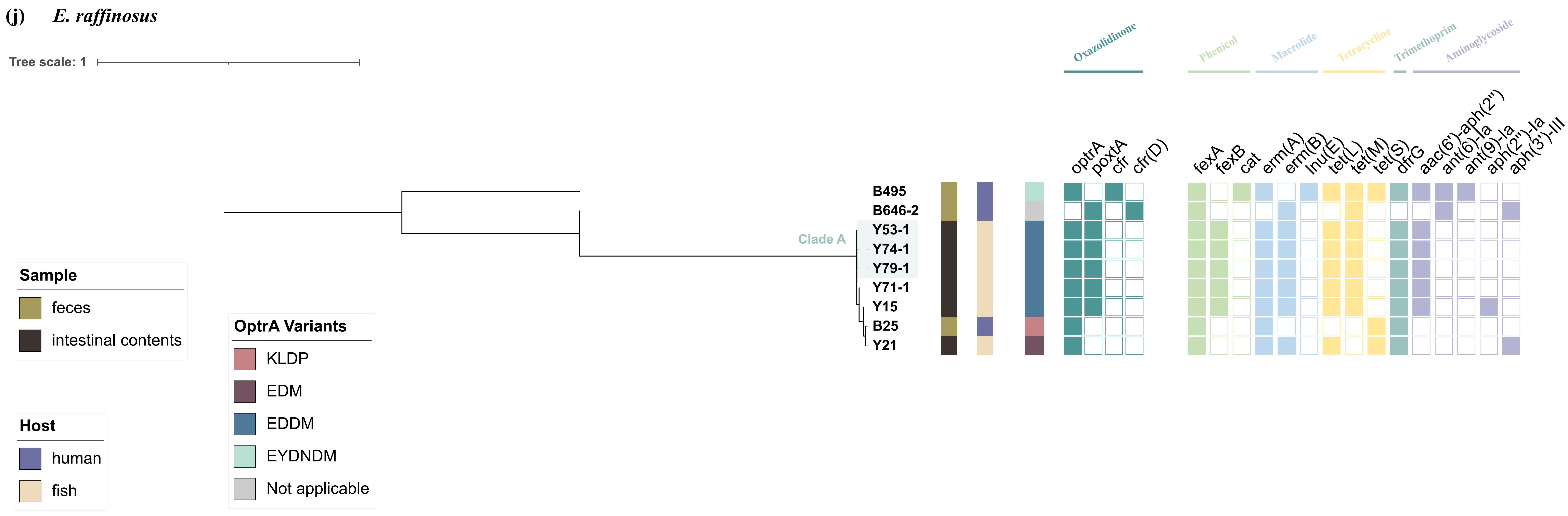
(j) E. raffinosus
Tree scale: 1
Oxazolidinone
Phenicol
Macrolide
Tetracycline
Trimethoprim
Aminoglycoside
optrA
poxtA
cfr
cfr(D)
fexA
fexB
cat
erm(A)
erm(B)
lnu(E)
tet(L)
tet(M)
tet(S)
dfrG
aac(6')-aph(2'')
ant(6)-Ia
ant(9)-Ia
aph(2'')-Ia
aph(3')-III
B495
B646-2
Clade A
Y53-1
Y74-1
Y79-1
Y71-1
Y15
B25
Y21
Sample
feces
intestinal contents
OptrA Variants
KLDP
EDM
EDDM
EYDNDM
Not applicable
Host
human
fish

Figure S2

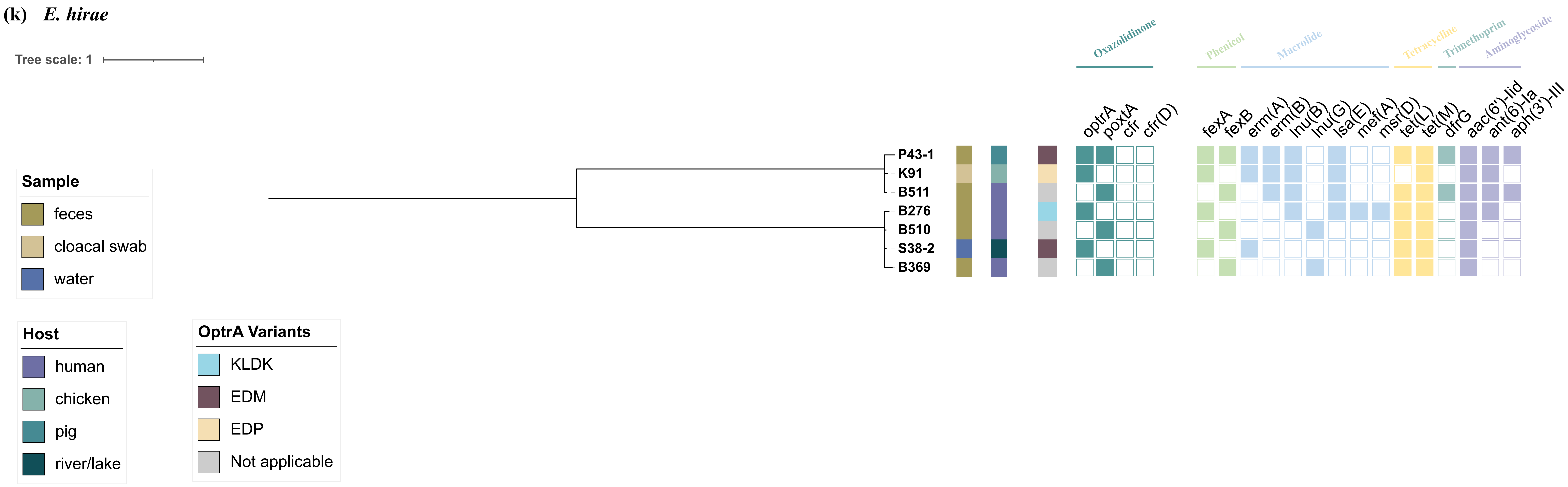
(k) E. hirae
Tree scale: 1
Oxazolidinone
Phenicol
Macrolide
Tetracycline
Trimethoprim
Aminoglycoside
optrA
poxtA
cfr
cfr(D)
fexA
fexB
erm(A)
erm(B)
lnu(B)
lnu(G)
lsa(E)
mef(A)
msr(D)
tet(L)
tet(M)
dfrG
aac(6')-Iid
ant(6)-Ia
aph(3')-III
P43-1
K91
B511
B276
B510
S38-2
B369
Sample
feces
cloacal swab
water
Host
human
chicken
pig
river/lake
OptrA Variants
KLDK
EDM
EDP
Not applicable

Figure S2

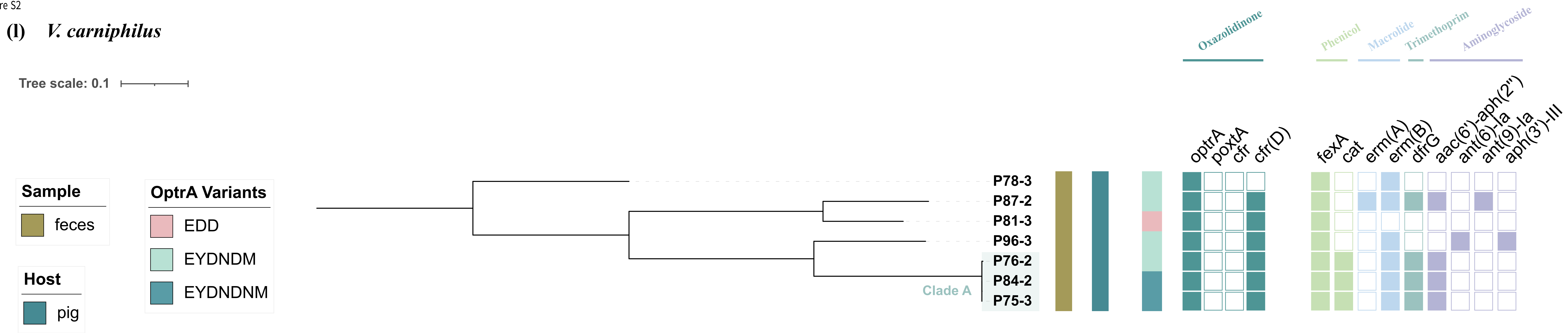
(l) V. carniphilus
Tree scale: 0.1
Sample
feces
Host
pig
OptrA Variants
EDD
EYDNDM
EYDNDNM
Clade A
P78-3
P87-2
P81-3
P96-3
P76-2
P84-2
P75-3
Oxazolidinone
Phenicol
Macrolide
Trimethoprim
Aminoglycoside
optrA
poxtA
cfr
cfr(D)
fexA
cat
erm(A)
erm(B)
dfrG
aac(6')-aph(2'')
ant(6)-Ia
ant(9)-Ia
aph(3')-III

Figure S2

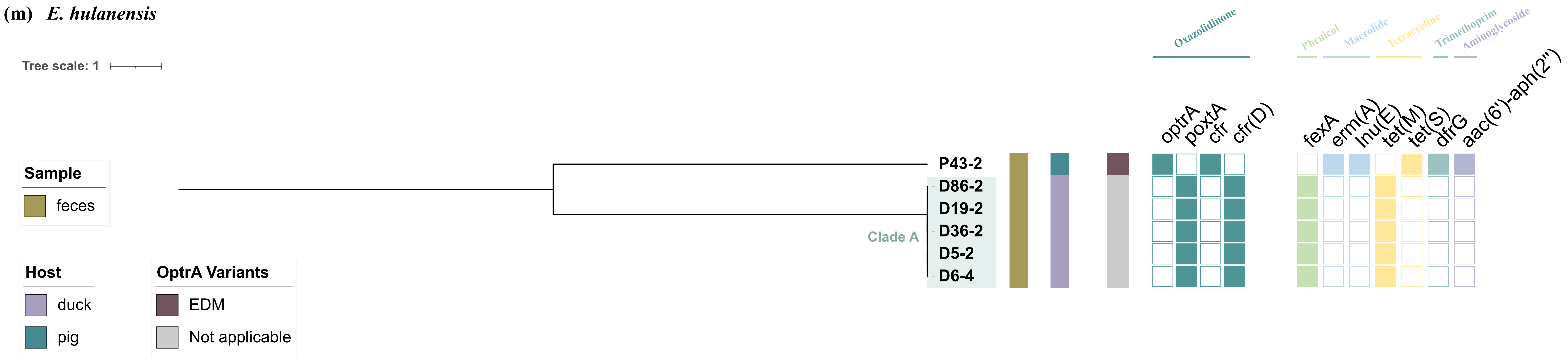
(m) E. hulanensis
Tree scale: 1
Sample
feces
Host
duck
pig
OptrA Variants
EDM
Not applicable
Clade A
P43-2
D86-2
D19-2
D36-2
D5-2
D6-4
Oxazolidinone
Phenicol
Macrolide
Tetracycline
Trimethoprim
Aminoglycoside
optrA
poxtA
cfr
cfr(D)
fexA
erm(A)
lnu(E)
tet(M)
tet(S)
dfrG
aac(6')-aph(2'')
