# Supplementary material for: Mapping the widespread distribution and transmission dynamics of linezolid resistance in humans, animals, and the environment
Source: Microbiome. 2024 Mar 13;12:52. doi: 10.1186/s40168-023-01744-2 (PMC10936028; doi:10.1186/s40168-023-01744-2)
Supplement: Supplementary file 5 — Additional file 4: Supplementary Figure S3. The genetic environment of the linezolid resistance genes. The arrows indicate the positions and directions of transcription of the different genes. All insertion sequences were labeled as orange, antimicrobial resistance genes were labeled as red, gene encoding proteins with known functions were labeled as blue, the rep genes were labeled as green, and the hypothesis proteins were labeled as grey. The letters at the lower right corner of optrA indicated the OptrA variants. N represented the number of strains that harbored the corresponding resistance locus encoding the gene or variants. Δ symbol indicates a truncated gene. [file 40168_2023_1744_MOESM4_ESM.pdf]

(a)

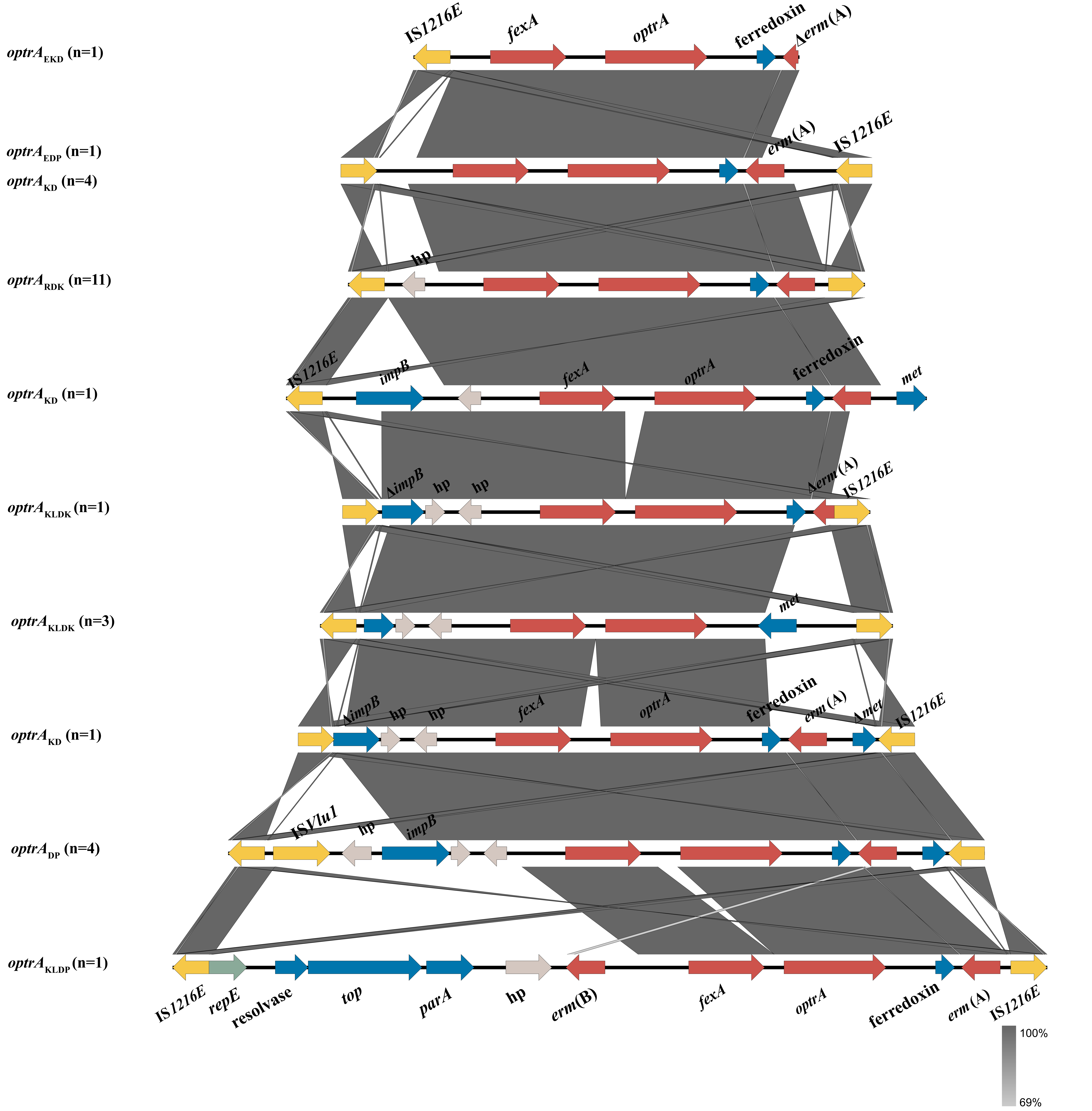

optrA EKD (n=1)
optrA EDP (n=1)
optrA KD (n=4)
optrA RDK (n=11)
optrA KD (n=1)
optrA KLDK (n=1)
optrA KLDK (n=3)
optrA KD (n=1)
optrA DP (n=4)
optrA KLDP (n=1)
IS1216E
fexA
optrA
ferredoxin
Δerm(A)
erm(A)
hp
impB
met
ΔimpB
Δmet
ISVlu1
repE
resolvase
top
parA
erm(B)
100%
69%

(b)

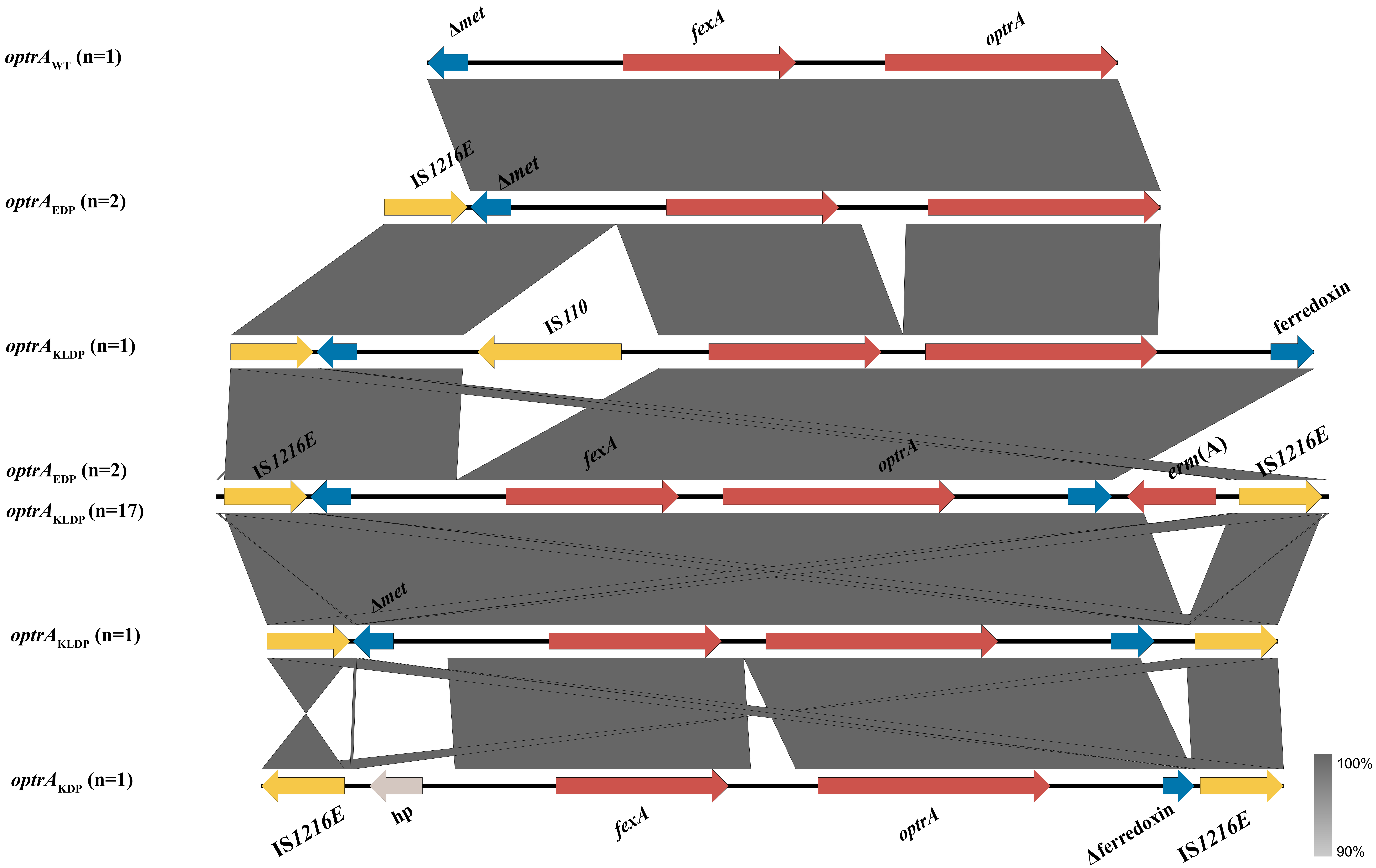

optrA$_{WT}$ (n=1)
Δmet
fexA
optrA
optrA$_{EDP}$ (n=2)
IS1216E
Δmet
optrA$_{KLDP}$ (n=1)
IS110
ferredoxin
optrA$_{EDP}$ (n=2)
optrA$_{KLDP}$ (n=17)
IS1216E
fexA
optrA
erm(A)
IS1216E
optrA$_{KLDP}$ (n=1)
Δmet
optrA$_{KDP}$ (n=1)
IS1216E
hp
fexA
optrA
Δferredoxin
IS1216E
100%
90%

(c)

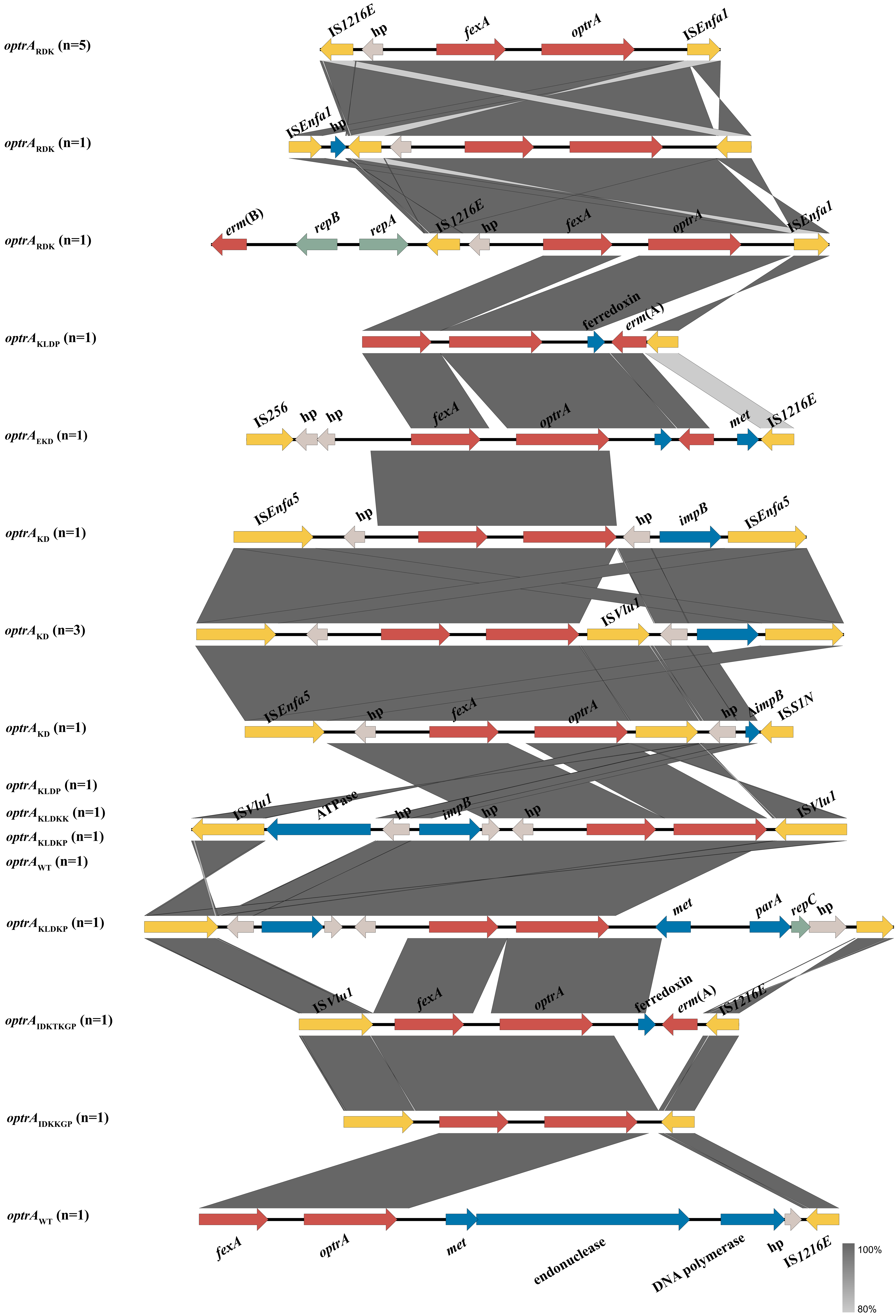

optrA_RDK (n=5)
IS1216E
hp
fexA
optrA
ISEnfa1
optrA_RDK (n=1)
ISEnfa1
hp
optrA_RDK (n=1)
erm(B)
repB
repA
IS1216E
hp
fexA
optrA
ISEnfa1
optrA_KLDP (n=1)
ferredoxin
erm(A)
optrA_EKD (n=1)
IS256
hp
hp
fexA
optrA
met
IS1216E
optrA_KD (n=1)
ISEnfa5
hp
hp
impB
ISEnfa5
optrA_KD (n=3)
ISVlu1
optrA_KD (n=1)
ISEnfa5
hp
fexA
optrA
hp
ΔimpB
ISS1N
optrA_KLDP (n=1)
optrA_KLDKK (n=1)
optrA_KLDKP (n=1)
optrA_WT (n=1)
ISVlu1
ATPase
hp
impB
hp
hp
ISVlu1
optrA_KLDKP (n=1)
met
parA
repC
hp
optrA_IDKTKGP (n=1)
ISVlu1
fexA
optrA
ferredoxin
erm(A)
IS1216E
optrA_IDKKGP (n=1)
optrA_WT (n=1)
fexA
optrA
met
endonuclease
DNA polymerase
hp
IS1216E
100%
80%

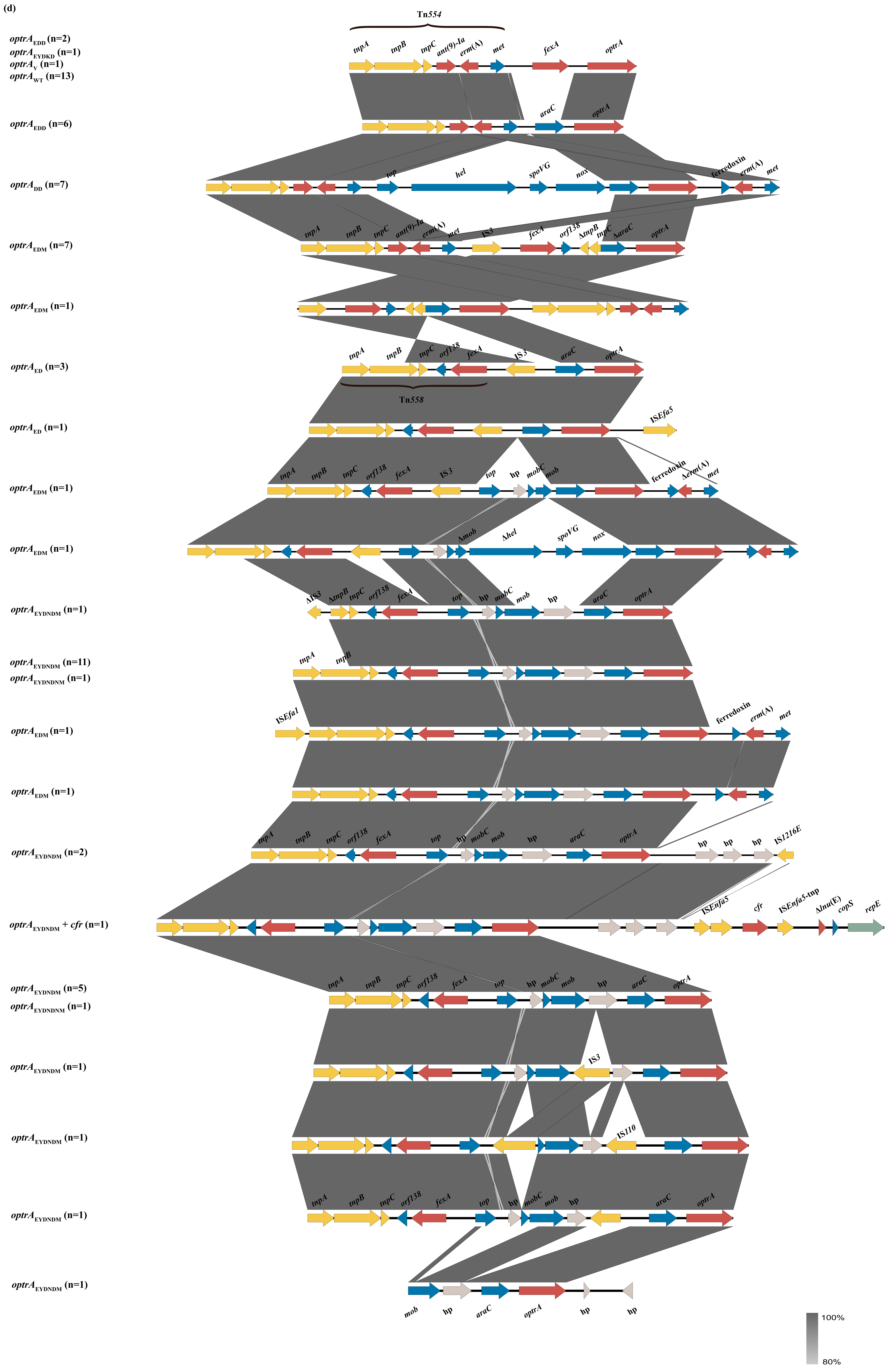

(d)
Tn554
optrA_EDD (n=2)
optrA_EYDKD (n=1)
optrA_V (n=1)
optrA_WT (n=13)
tnpA tnpB tnpC ant(9)-Ia erm(A) met fexA optrA
optrA_EDD (n=6)
araC optrA
optrA_DD (n=7)
top hel spoVG nox ferredoxin erm(A) met
optrA_EDM (n=7)
tnpA tnpB tnpC ant(9)-Ia erm(A) met IS3 fexA orf138 ΔtnpB tnpC ΔaraC optrA
optrA_EDM (n=1)
optrA_ED (n=3)
tnpA tnpB tnpC orf138 fexA IS3 araC optrA
Tn558
optrA_ED (n=1)
ISEfa5
optrA_EDM (n=1)
tnpA tnpB tnpC orf138 fexA IS3 top hp mobC mob ferredoxin Δerm(A) met
optrA_EDM (n=1)
Δmob Δhel spoVG nox
optrA_EYDNDM (n=1)
ΔIS3 ΔtnpB tnpC orf138 fexA top hp mobC mob hp araC optrA
optrA_EYDNDM (n=11)
optrA_EYDNDNM (n=1)
tnpA tnpB
optrA_EDM (n=1)
ISEfa1 ferredoxin erm(A) met
optrA_EDM (n=1)
optrA_EYDNDM (n=2)
tnpA tnpB tnpC orf138 fexA top hp mobC mob hp araC optrA hp hp hp IS1216E
optrA_EYDNDM + cfr (n=1)
ISEnfa5 cfr ISEnfa5-tnp Δlnu(E) copS repE
optrA_EYDNDM (n=5)
optrA_EYDNDNM (n=1)
tnpA tnpB tnpC orf138 fexA top hp mobC mob hp araC optrA
optrA_EYDNDM (n=1)
IS3
optrA_EYDNDM (n=1)
IS110
optrA_EYDNDM (n=1)
tnpA tnpB tnpC orf138 fexA top hp mobC mob hp araC optrA
optrA_EYDNDM (n=1)
mob hp araC optrA hp hp
100%
80%

(e)

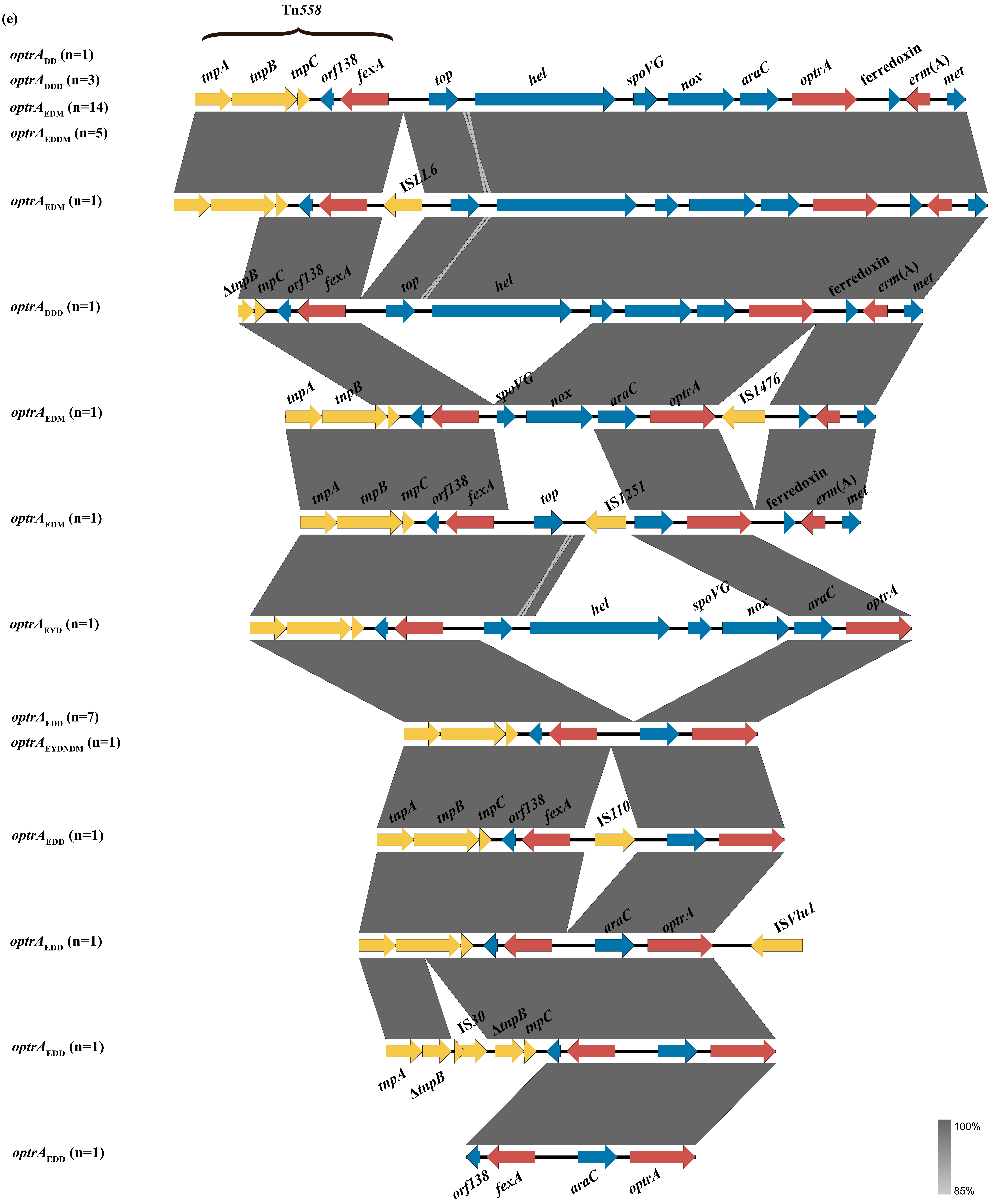
Tn558
optrA_DD (n=1)
optrA_DDD (n=3)
optrA_EDM (n=14)
optrA_EDDM (n=5)
tnpA
tnpB
tnpC
orf138
fexA
top
hel
spoVG
nox
araC
optrA
ferredoxin
erm(A)
met
optrA_EDM (n=1)
ISLL6
optrA_DDD (n=1)
ΔtnpB
optrA_EDM (n=1)
IS1476
optrA_EDM (n=1)
IS1251
optrA_EYD (n=1)
optrA_EDD (n=7)
optrA_EYDNDM (n=1)
optrA_EDD (n=1)
IS110
optrA_EDD (n=1)
ISVlu1
optrA_EDD (n=1)
IS30
optrA_EDD (n=1)
100%
85%

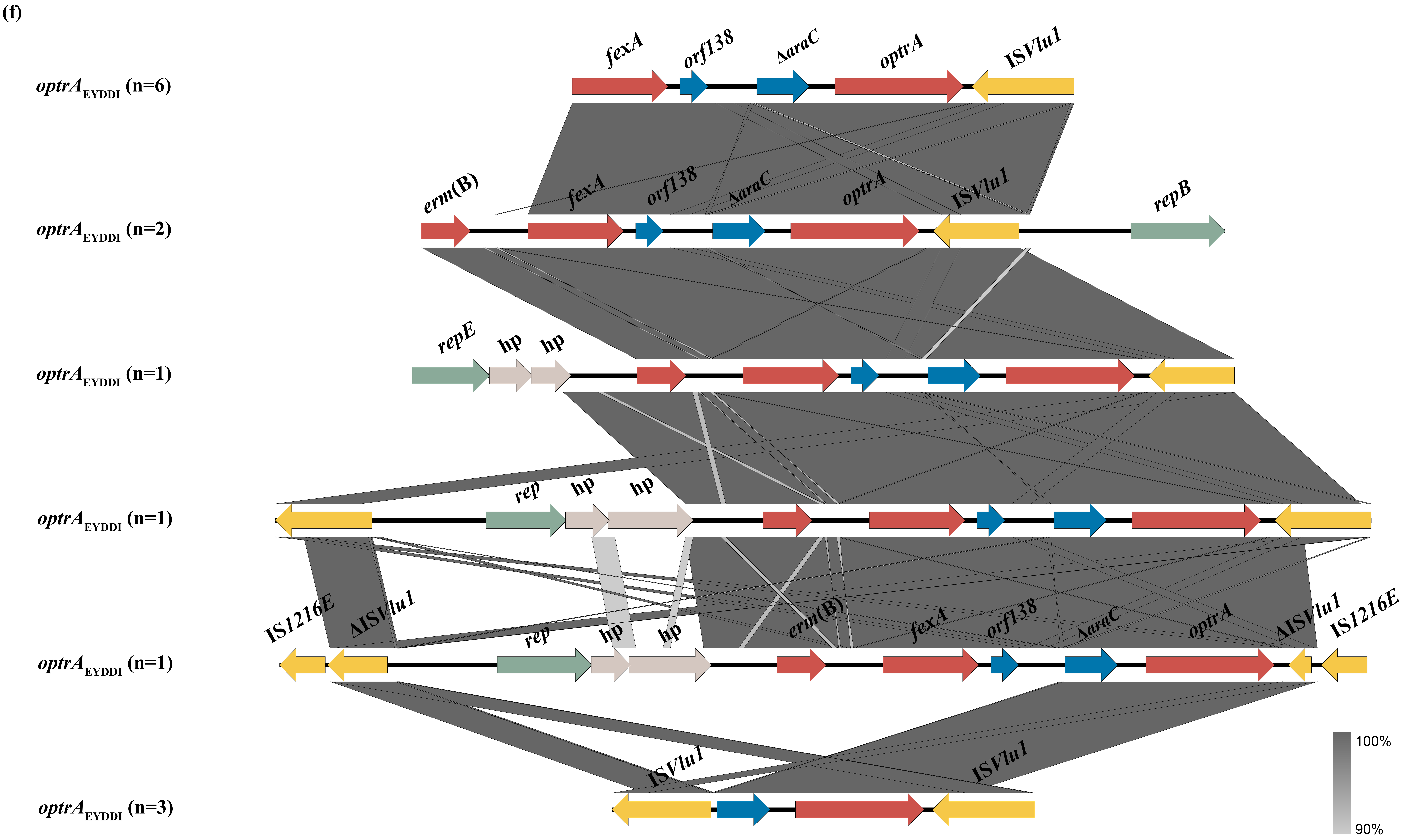
(f)
optrA EYDDI (n=6)
fexA
orf138
ΔaraC
optrA
ISVlu1
optrA EYDDI (n=2)
erm(B)
fexA
orf138
ΔaraC
optrA
ISVlu1
repB
optrA EYDDI (n=1)
repE
hp
hp
optrA EYDDI (n=1)
rep
hp
hp
optrA EYDDI (n=1)
IS1216E
ΔISVlu1
rep
hp
hp
erm(B)
fexA
orf138
ΔaraC
optrA
ΔISVlu1
IS1216E
optrA EYDDI (n=3)
ISVlu1
ISVlu1
100%
90%

(g)

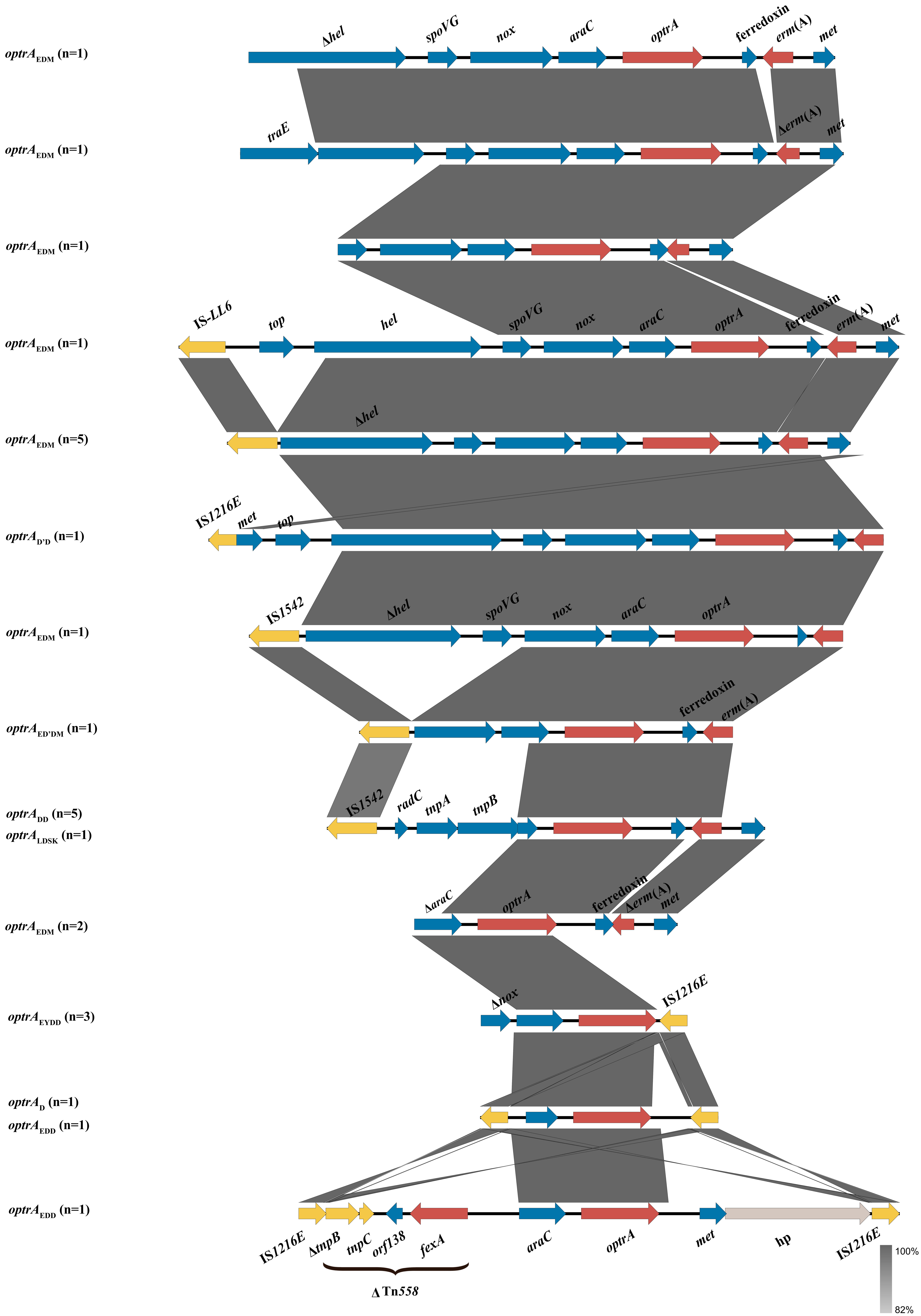

optrA_EDM (n=1)
Δhel
spoVG
nox
araC
optrA
ferredoxin
erm(A)
met
optrA_EDM (n=1)
traE
Δerm(A)
met
optrA_EDM (n=1)
optrA_EDM (n=1)
IS-LL6
top
hel
spoVG
nox
araC
optrA
ferredoxin
erm(A)
met
optrA_EDM (n=5)
Δhel
optrA_D'D (n=1)
IS1216E
met
top
optrA_EDM (n=1)
IS1542
Δhel
spoVG
nox
araC
optrA
optrA_ED'DM (n=1)
ferredoxin
erm(A)
optrA_DD (n=5)
optrA_LDSK (n=1)
IS1542
radC
tnpA
tnpB
optrA_EDM (n=2)
ΔaraC
optrA
ferredoxin
Δerm(A)
met
optrA_EYDD (n=3)
Δnox
IS1216E
optrA_D (n=1)
optrA_EDD (n=1)
optrA_EDD (n=1)
IS1216E
ΔtnpB
tnpC
orf138
fexA
araC
optrA
met
hp
IS1216E
Δ Tn558
100%
82%

(h)

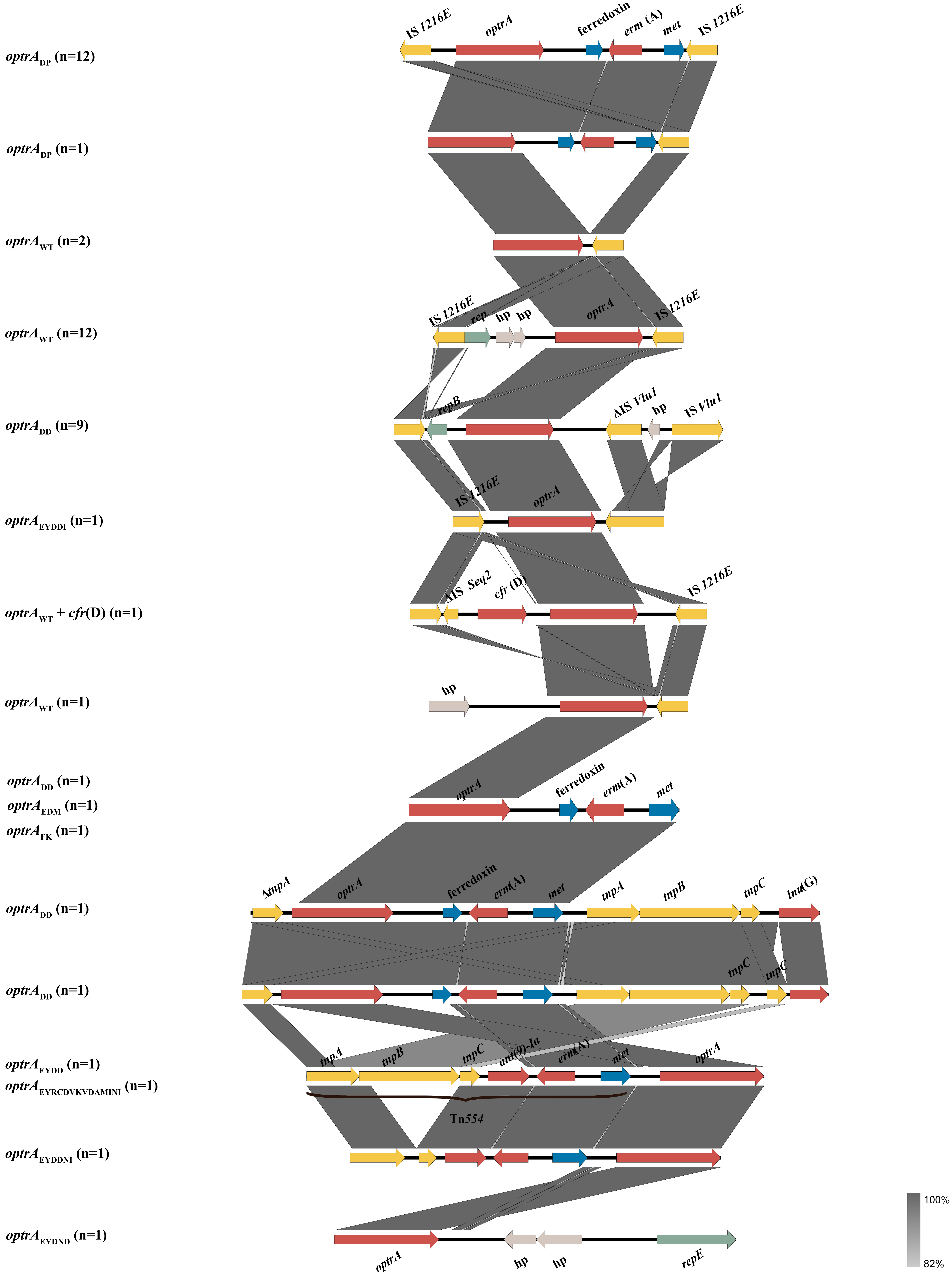

optrA_DP (n=12)
optrA_DP (n=1)
optrA_WT (n=2)
optrA_WT (n=12)
optrA_DD (n=9)
optrA_EYDDI (n=1)
optrA_WT + cfr(D) (n=1)
optrA_WT (n=1)
optrA_DD (n=1)
optrA_EDM (n=1)
optrA_FK (n=1)
optrA_DD (n=1)
optrA_DD (n=1)
optrA_EYDD (n=1)
optrA_EYRCDVKVDAMINI (n=1)
optrA_EYDDNI (n=1)
optrA_EYDND (n=1)
IS 1216E
optrA
ferredoxin
erm (A)
met
rep
hp
repB
ΔIS Vlu1
IS Vlu1
ΔIS Seq2
cfr (D)
ΔtnpA
tnpA
tnpB
tnpC
lnu(G)
ant(9)-Ia
Tn554
repE
100%
82%

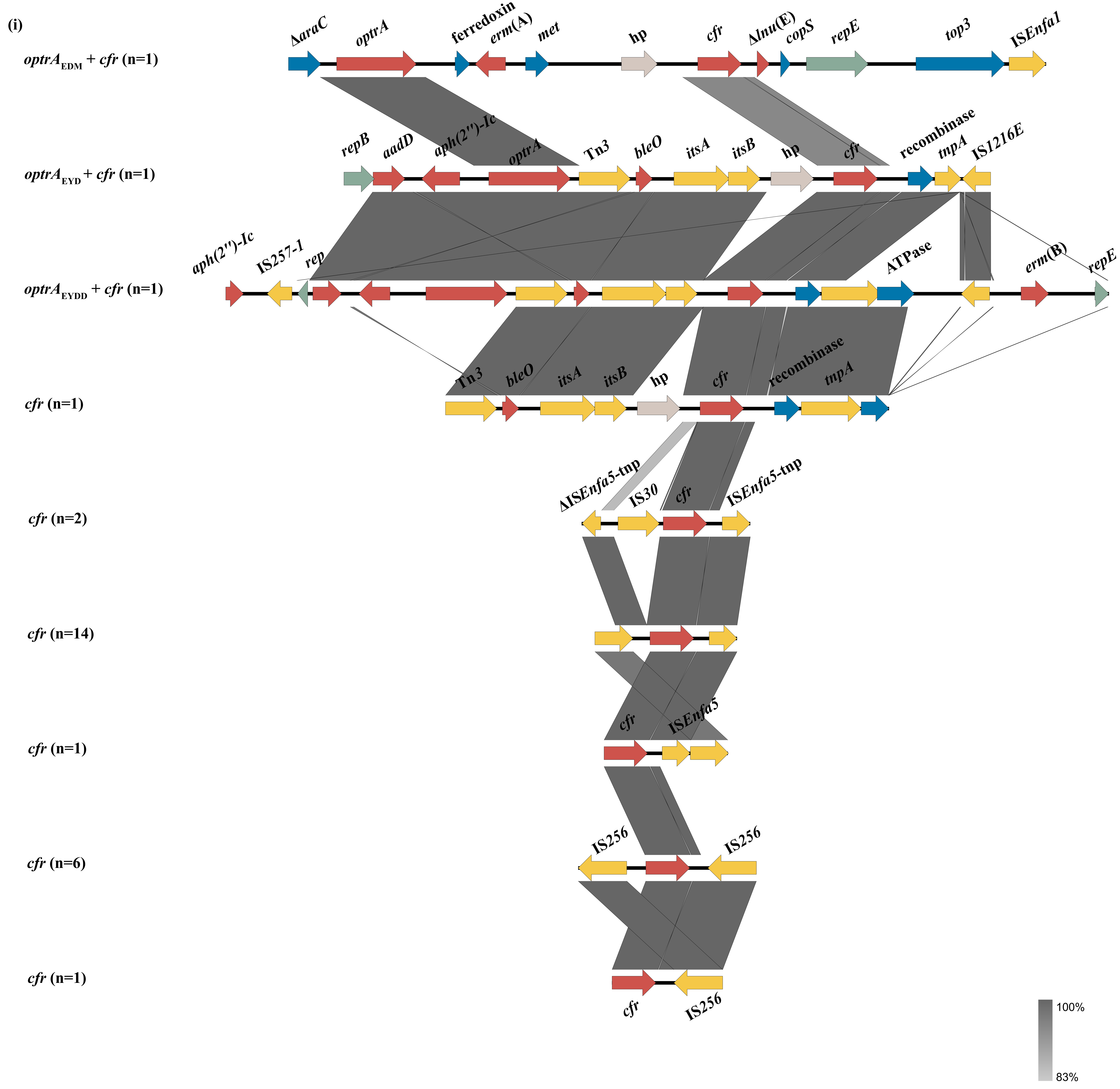
(i)
optrA_EDM + cfr (n=1)
ΔaraC
optrA
ferredoxin
erm(A)
met
hp
cfr
Δlnu(E)
copS
repE
top3
ISEnfa1
optrA_EYD + cfr (n=1)
repB
aadD
aph(2'')-Ic
optrA
Tn3
bleO
itsA
itsB
hp
cfr
recombinase
tnpA
IS1216E
optrA_EYDD + cfr (n=1)
aph(2'')-Ic
IS257-1
rep
ATPase
erm(B)
repE
cfr (n=1)
Tn3
bleO
itsA
itsB
hp
cfr
recombinase
tnpA
cfr (n=2)
ΔISEnfa5-tnp
IS30
cfr
ISEnfa5-tnp
cfr (n=14)
cfr (n=1)
cfr
ISEnfa5
cfr (n=6)
IS256
IS256
cfr (n=1)
cfr
IS256
100%
83%

(j)

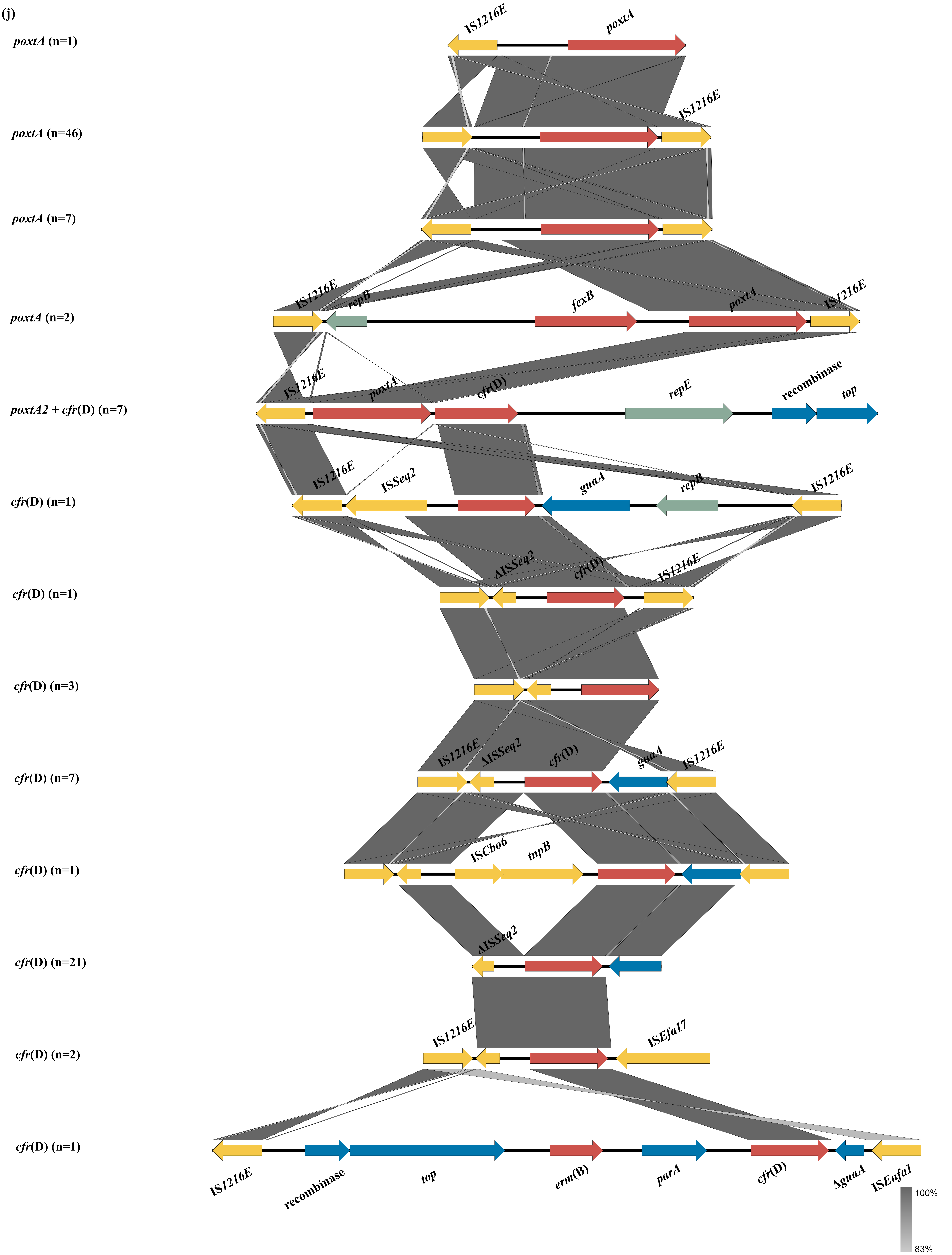

poxtA (n=1)
poxtA (n=46)
poxtA (n=7)
poxtA (n=2)
poxtA2 + cfr(D) (n=7)
cfr(D) (n=1)
cfr(D) (n=1)
cfr(D) (n=3)
cfr(D) (n=7)
cfr(D) (n=1)
cfr(D) (n=21)
cfr(D) (n=2)
cfr(D) (n=1)
IS1216E
poxtA
repB
fexB
cfr(D)
repE
recombinase
top
ISSeq2
guaA
ΔISSeq2
ISCbo6
tnpB
ISEfa17
erm(B)
parA
ΔguaA
ISEnfa1
100%
83%
